# Ultra-Processed Foods and Health Outcomes: A Narrative Review

**DOI:** 10.3390/nu12071955

**Published:** 2020-06-30

**Authors:** Leonie Elizabeth, Priscila Machado, Marit Zinöcker, Phillip Baker, Mark Lawrence

**Affiliations:** 1 School of Exercise and Nutrition Science, Deakin University, Geelong 3217, Australia; lelizabe@deakin.edu.au (L.E.); p.machado@deakin.edu.au (P.M.); phil.baker@deakin.edu.au (P.B.); 2 Institute for Physical Activity and Nutrition, Deakin University, Geelong 3217, Australia; 3 Department of Nutrition, Bjørknes University College, 0456 Oslo, Norway; marit.zinocker@bhioslo.no

**Keywords:** ultra-processed food, health outcomes, dietary patterns, NOVA, food processing, obesity

## Abstract

The nutrition literature and authoritative reports increasingly recognise the concept of ultra-processed foods (UPF), as a descriptor of unhealthy diets. UPFs are now prevalent in diets worldwide. This review aims to identify and appraise the studies on healthy participants that investigated associations between levels of UPF consumption and health outcomes. This involved a systematic search for extant literature; integration and interpretation of findings from diverse study types, populations, health outcomes and dietary assessments; and quality appraisal. Of 43 studies reviewed, 37 found dietary UPF exposure associated with at least one adverse health outcome. Among adults, these included overweight, obesity and cardio-metabolic risks; cancer, type-2 diabetes and cardiovascular diseases; irritable bowel syndrome, depression and frailty conditions; and all-cause mortality. Among children and adolescents, these included cardio-metabolic risks and asthma. No study reported an association between UPF and beneficial health outcomes. Most findings were derived from observational studies and evidence of plausible biological mechanisms to increase confidence in the veracity of these observed associations is steadily evolving. There is now a considerable body of evidence supporting the use of UPFs as a scientific concept to assess the ‘healthiness’ of foods within the context of dietary patterns and to help inform the development of dietary guidelines and nutrition policy actions.

## 1. Introduction

The concept of ultra-processed food (UPF) as a descriptor of unhealthy foods within dietary patterns is increasingly recognised in the nutrition literature [1,2,3,4,5] and authoritative reports [6,7]. Understanding of the contribution of UPFs to dietary quality and as a risk factor for diet-related diseases, disorders and conditions is rapidly emerging [8]. Yet, limited consideration has been given to UPF in strategies aiming to improve population health [9]. A crucial missing step in closing that gap is a review of the evidence base of the associations between UPF consumption and adverse health outcomes. 

Dietary risk factors are leading contributors to the global burden of disease (GBD), responsible for an estimated 11 million deaths from non-communicable diseases (NCDs) (22% of all adult deaths) and 15% of disability life years (DALYs) lost in 2017 [10]. Leading contributors to diet-related deaths are cardiovascular disease (CVD), cancer and type 2 diabetes [10]. Contributors to DALYs from non-fatal chronic conditions include asthma, musculoskeletal conditions and mental health disorders [11].

Implicated dietary risk factors include certain nutrients, foods and dietary pattern exposures. Nutrient exposures include high amounts of sodium [10,12], saturated fat, trans-fat and added sugar [12]. Food exposures include low amounts of whole grains, fruit, vegetables, nuts and seeds [10] and fish [10,12], and high amounts of red meat, processed meat, potato chips and sugar-sweetened beverages (SSB) [12,13]. Dietary patterns include low scores on the Healthy Eating Index or Alternative Healthy Eating Index [14], or Mediterranean Dietary Pattern [15]; low adherence to the Dietary Approaches to Stop Hypertension diet [16]; or a high score on the Western dietary pattern [17,18,19,20].

In a novel approach to food categorization, NOVA (a name not an acronym) classifies foods and beverages ‘according to the extent and purpose of industrial processing’ [21,22], an aspect generally overlooked by public health nutrition science, policy and guidance. In 2009, a Brazilian research group, following studies on national trends over 25 years on household food acquisition and health implications [23,24], concluded diets containing high proportions of UPFs are intrinsically nutritionally unbalanced, harmful to health, or both [9]. This led to the development of the NOVA food classification system [25], which has since evolved [21,22,26,27,28,29,30].

The NOVA classification assigns foods to one of four groups, based on ‘the extent and purpose of industrial processing’ [21]: (1) ‘unprocessed or minimally processed foods’ (MPF), comprising edible parts of plants, animals or fungi without any processes applied to them or natural foods altered by minimal processing designed to preserve natural foods to make them suitable for storage, or to make them safe, edible or more palatable (e.g., fresh fruit, vegetables, grains, legumes, meat, milk); (2) processed culinary ingredients (PCI), which are substances extracted from group 1 (e.g., fats, oils, sugars and starches) or from nature (e.g., salt) used to cook and season MPF, not intended for consumption on their own; (3) processed foods (PF), where industrial products are made by adding PCI to MPF (e.g., canned vegetables in brine, fruit in syrup, cheese); and (4) UPFs, which are defined as ‘formulations of ingredients, mostly of exclusive industrial use, that result from a series of industrial processes (hence “ultra-processed”), many requiring sophisticated equipment and technology’ (e.g., sweet and savoury snacks, reconstituted meats, pizza dishes and confectionery, among others) [21]. Ingredients characteristic of UPFs include food substances of no or rare culinary use, including sugar, protein and oil derivatives (e.g., high-fructose corn syrup, maltodextrin, protein isolates, hydrogenated oil) and cosmetic additives (e.g., colours, flavours, flavour enhancers, emulsifiers, thickeners, and artificial sweeteners) designed to make the final product more palatable [21].

Since NOVA was established, nutrition researchers worldwide have increasingly implicated UPFs with poor dietary quality, and with adverse metabolic and health outcomes across a range of populations and country contexts [7]. Furthermore, UPFs have become dominant components in diets of populations worldwide [31], contributing up to more than 50% of energy intake in high-income countries [32,33], and up to 30% in middle-income countries [34,35], with consumption volumes rapidly increasing [36,37,38]. Because middle-income countries are home to the vast bulk of the world’s population, understanding the implications of rising UPF consumption for global human health is of utmost importance.

Several reviews have reported on UPFs and health outcomes [2,3,4,5,7,39]. However, despite the large and rapidly growing body of evidence linking UPFs with adverse health outcomes, the number of reviews and summarizing reports to date have been scarce, possibly delaying the inclusion of the ‘extent and purpose of industrial processing’ [21] as an independent factor for assessing the health potential of diets. As most dietary advice relies on systematic reviews and meta-analyses when reviewing evidence, a comprehensive review could be helpful in strengthening the evidence base and moving this field forward. To our knowledge, no review to date has employed a systematic search to identify all studies, without the restriction of health outcomes or study design.

The aim of this narrative review was to systematically identify and appraise the findings of studies on healthy participants (adults, adolescents and children) that have investigated associations between levels of UPF consumption and health outcomes.

## 2. Materials and Methods

A systematic search and narrative review method were adopted [40,41], involving four main steps: first, a systematic search process and application of inclusion and exclusion criteria; second, data extraction and synthesis of results; third, an analysis of key findings by narrative review; fourth, a quality appraisal procedure that included all studies. The method allowed a thorough search for extant literature, integration and interpretation of findings from diverse study types, populations, health outcomes, measurements and dietary assessments as well as quality appraisal. Checklists were used to ensure thoroughness of relevant components of the narrative review and systematic methods [42,43,44].

### 2.1. Search Process

**Figure 1:**
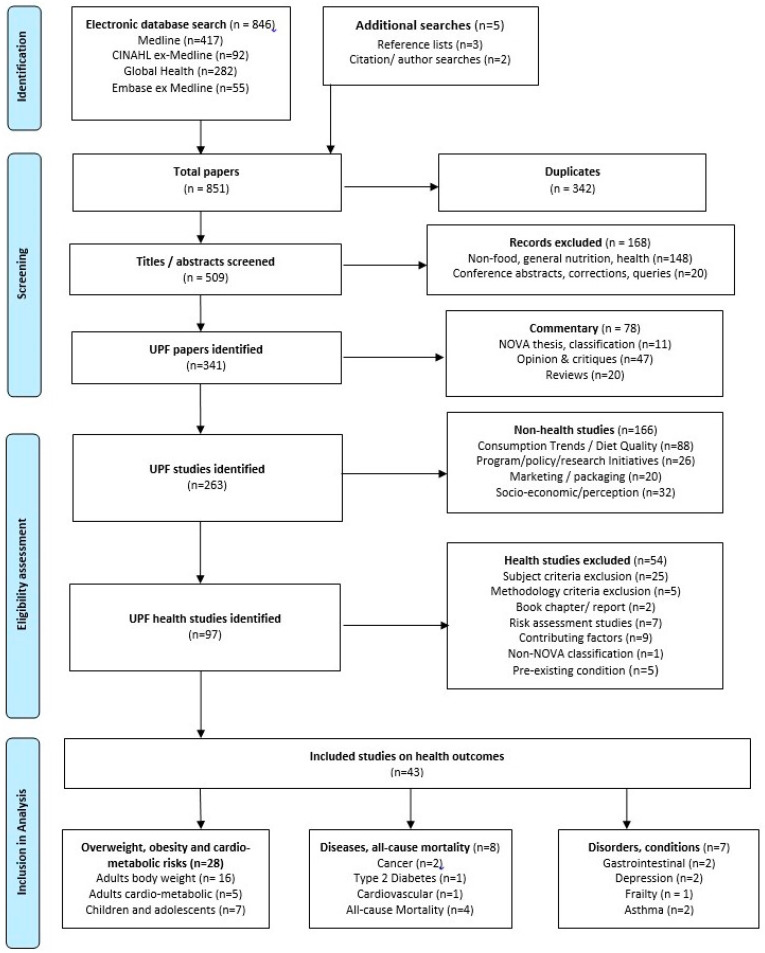
Flow Diagram of Database Search and Article Eligibility (modified from PRISMA flow diagram [43,44]).

The study utilised a systematic search process to ensure relevant studies were retrieved. Searches were performed in July 2019 using four databases—Medline, CINAHL, Global Health and Embase—and searching string variations of the following keywords: (ultra-process* or ultra process* or ultraprocess*) or (NOVA and “food classification”). Google Scholar citation searches and manual searches of reference lists were also conducted to identify relevant studies. A further EBSCO-host combined search was undertaken to identify any literature reviews and meta-analyses potentially missed by the initial searches. Additional searches of the electronic databases were performed in February 2020 and of Medline on 21 May 2020, to identify studies published in the interim. Records were downloaded to EndNote X8.2, and duplicates removed. Titles were first screened by title and abstract, then by full text against the inclusion and exclusion criteria given in Table 1. Eligible articles were studies evaluating exposure (defined as ‘levels of consumption’) to ‘ultra-processed foods’ (defined as group 4 of the NOVA food classification system [21], described in Supplementary Material Table S1) and a ‘health outcome’ (defined as any disease, disorder or condition specified in the International Classification of Diseases, 11th Revision [45]). In this review, ‘food’ includes foods and beverages; ‘consumption’ includes national or household food availability and individual food intake. From a total of 851 papers, there were 263 identified studies on UPF and 43 for final inclusion in our review. Figure 1 presents a flow diagram adapted from PRISMA [43,44] of the search process.

### 2.2. Data Extraction, Synthesis, Analysis and Quality Appraisal

Data were extracted on study details (author, publication date, study type, country and period of study) population (subjects, sample size), UPF exposure (food data extraction level, collection method, relative exposure assessment, NOVA reference), health outcomes (definition, data collection) and key findings. Studies were organised and tabulated into three classifications by specified health outcomes: (1) overweight, obesity and cardio-metabolic risks (hereafter referred to as ‘risks’), with ‘overweight and obesity’ also encompassing BMI, weight gain and related factors such as body fat percentage, body fat distribution, waist circumference; and ‘cardio-metabolic risks’ including high blood pressure, metabolic syndrome and relevant biomarkers for cardiovascular disease or type 2 diabetes; (2) diseases including cancer, cardiovascular disease and type 2 diabetes, and mortality (hereafter, referred to as ‘diseases’); and (3) other disorders and conditions (hereafter referred to as ‘disorders’). Studies on children and adolescents were considered separately to those on adults. In one study results on adults and adolescents were reported separately.

Data were extracted for the associations adjusted for potential confounders. Crude associations were extracted when the adjusted analysis was not performed. We presented only statistically significant associations (hereafter termed ‘associations’) in the written results and the relative risk of UPF exposure. For studies with a prospective design that also presented cross-sectional data, we presented the prospective results. There were varying terminologies describing body weight in the studies. We presented authors’ terminologies in Table 2 and Table 3. In the results and discussion, we standardised to World Health Organization (WHO) definitions and BMI (kg/m^2^) cut-off brackets for adults—(1) ‘overweight’ (BMI ≥ 25), to clarify this includes obesity; (2) ‘overweight (BMI 25–30)’ (BMI = 25.0–29.9), to clarify this excludes obesity; and (3) ‘obesity’ (BMI ≥ 30)—and WHO BMI-for-age z-scores for children, unless otherwise stated [46,47,48]. When more than one reference was included as NOVA reference, we assumed the most recent was applied to data classification.

The analysis was addressed by a narrative review with a comprehensive exploration of general direct and negative associations in different health outcomes, associations within studies, differential effects of study types, populations and ages, exposure differences, and outcome measures. Objectivity was achieved by a quality assurance process, including group consensus; and for each article, a quality assessment was undertaken [49,50,51], as described in Supplementary Material Table S3.

**Table 2:** Overweight, obesity and cardio-metabolic risks as outcomes in studies in adults *.

Study Details			UPF Exposure			Outcomes		Results
Publication Author(s) Year	Study Type (Year) Setting	Population (Number)	Extraction Level	Relative exposure [UPF Reference Year]	Data Collection Method	Health Outcome(s) (Study Definition)	Data Collection Method	Key Findings
**Overweight and obesity**								
Juul 2015 [36]	Ecological (1960–2010) Sweden	Adults ≥18 years (*n* = −4000 household)	National + household sampling	*National*: per capita UPF consumption + *Household*: UPF % share food purchase (kg or litre per capita per annum) [NOVA.2014] [29]	*National*: Swedish BOA net food ** available *Household*: 2-week purchase record by interview	BMI classified in prevalence overweight (BMI ≥ 25) and obesity (BMI ≥ 30)	National population statistics	From 1980 to 2008: rise in overweight prevalence for men from 35% to 54–56% and women from 26% to 39%; and obesity prevalence for men rose from 4.5% to 11% and for women from 5% to 10%. From 1960 to 2010 rise in UPF consumption of 142% tracks increase in overweight and obesity prevalence.
Monteiro 2017 [52]	Ecological (1991–2008) Europe	Adults ≥ 18 years except Belgium ≥ 15 years (*n* = 19 countries)	Household (National Sample)	UPF % total E purchases (continuous) [NOVA.2018] [30]	Belgium, Sweden, Germany = one month food ** purchase record; all others = 14 day record g/mL.	BMI classified in prevalence obesity (BMI ≥ 30)	National reports	UPF ranged 10.2–50.7% (median 26.4) of household total E in food purchases. Each 1% increase in UPF E availability was associated with 0.25% increase in obesity prevalence.
Vandevijvere 2019 [37]	Ecological (Repeated cross-sectional) (2002–2014) Global	Adults ≥18 years (*n* = 80 countries)	National	UPF total sales (volume/capita) [NOVA.2018] [30]	Volume sales of UPF (137 items from 212 food ** subgroups)	Mean population BMI	National reports	Increases in UPF volume sales/capita were directly associated with mean BMI trajectories. Every standard deviation increase in volume sales of UPF, mean BMI increased by 0.195 kg/m^2^ for men and 0.072 kg/m^2^ for women (drinks only), and 0.316 kg/m^2^ for men (foods only).
Canella 2014 [53]	Cross-sectional (2008–2009) Brazil	All ages (*n* = 55,970 households; 190,159) individuals)	Household (National Sample)	UPF % total E purchases (quartiles) [NOVA.2012] [54]	7-day food ** purchase record	BMI classified in excess weight (BMI > 25), obesity (BMI > 30) WHO BMI for age Z scores [children]	Trained personnel	UPF contributed 25.5% of total E purchased. Participants living in household strata belonging to the upper quartile of UPF consumption had higher mean BMI (Z score) (β = 0.19; 95% CI 0.14, 0.25) prevalence of obesity (β = 3.72; 95% CI 2.50, 4.94) and prevalence of excess weight (β = 6.27; 95% CI 4.15, 8.39), compared with those in the lowest quartile. As UPF consumption rose from Quartile 1 to Quartile 4, the prevalence of excess weight rose from 34.1% to 43.9%, and prevalence of obesity rose from 9.8% to 13.1%.
Adams 2015 [55]	Cross-sectional (2008–2012) UK	Adults > 18 years (*n* = 2174)	Individual (National Sample)	UPF % total E intake (continuous) [NOVA.2010] [25]	4-day food ** intake diary	BMI classified in overweight (BMI ≥ 25); obesity (BMI ≥ 30)	Trained personnel	UPF contributed 53% of total E intake. UPF consumption was not significantly associated with BMI, overweight and obesity, and obesity.
Louzada ‡ 2015 [56]	Cross-sectional (2008–2009) Brazil	Adults > 20 years; children > 10 years (*n* = 30,243)	Individual (National Sample)	UPF % total E intake (quintiles) [NOVA.2012] [54]	2 × 24-h food ** intake record	BMI classified in excess weight (BMI ≥ 25), obesity (BMI ≥ 30) [adults]; WHO BMI for age Z scores [children]	Trained personnel	UPF contributed to 29.6% of total E intake. Individuals in the upper quintile of UPF intake had significantly higher BMI (0.94 kg/m^2^; 95% CI = 0.42, 1.47) and higher odds of being obese (OR = 1.98; 95% CI = 1.26, 3.12) compared with the lowest quintile. No significant association with excess weight was found.
Nardocci 2018 [57]	Cross-sectional (2004–2005) Canada	Adults > 18 years (19,363)	Individual (National Sample)	UPF % total E intake (quintiles, and continuous) [NOVA2016.2018] [30,58]	1 × 24-h recall	BMI classified in overweight (25.0 ≤ BMI < 30.0); obesity (BMI ≥ 30)	Trained personnel	UPF contributed 45.1% of total E intake. Individuals in highest quintile UPF intake significantly had higher odds of being obese (OR = 1.32, 95% CI 1.05, 1.57, and overweight (OR = 1.03; 95% CI 1.01, 1.07), compared with individuals in lowest quintile.
Juul 2018 [59]	Cross-sectional (2005–2014) USA	Adults 20–64 years (15,977)	Individual (National sample)	UPF % total E intake (quintiles) [NOVA.2014] [29]	2 available 24-h recall or 1 day otherwise	BMI classified in overweight and obesity (BMI ≥ 25), obesity (BMI ≥ 30); WC classified in abdominal obesity (AO) [men ≥ 102 cm, women ≥ 88 cm)	Trained personnel	UPF contributed 56.1% of total E intake. Individuals in the highest quintile of UPF intake had significantly higher BMI (1.61 kg/m²; 95% CI 1.11, 2.10), and WC (4.07 cm, 95% CI 2.94, 5.19), and higher odds of having excess weight (OR = 1.48; 95% CI 1.25 to 1.76), obesity (OR = 1.53, 95% CI 1.29, 1.81), and abdominal obesity (OR = 1.62; 95% CI 1.39 to 1.89) compared with those in the lowest quintile.
Rauber 202 [60]	Cross-sectional (2008–2016) UK	Adults 19−96 years (*n* = 6143)	Individual (National sample)	UPF % total E intake (quartiles) [NOVA.2019] [21]	4-day food ** intake diary	BMI classified in obesity (BMI ≥ 30). WC classified in AO	Trained personnel	UPF contributed 54.3% of total E intake. Individuals in the highest quartile of UPF intake had higher BMI (1.66 kg/m2; 95%CI 0.96, 2.36) and WC (3.56cm, 95% CI 1.79, 5.33), and higher odds of obesity (OR = 1.90, 95% CI 1.39, 2.61) compared with the lowest quartile.
Julia 2018 [61]	Cross-sectional (2014) France	Adults Mean 43.8 years (*n* = 74,470)	Individual	UPF % total grams (quartiles) [NOVA.2016] [22,33]	3 × 24 h records	BMI classified in overweight (25–29.9), obesity (≥30)	Self-report #	UPF contributed 18.4% of total weight intake, and 35.9% of total E intake. Higher consumption of UPF by % E intake was independently associated with overweight (p < 0.0001); and higher intake by energy-weighted UPF was independently associated with overweight, and obesity (both *p* < 0.0001).
Silva 2018 [62]	Cross-sectional (2008–2010) Brazil	Active and retired civil servants 35–64 years (*n* = 8977)	Individual	UPF % total E intake (quartiles) [NOVA.2016] [22]	114 item-FFQ	BMI classified in overweight (25.0-29.9); obesity (≥30); WC classified in increased WC (men ≥ 94; women ≥ 80); significantly increased WC (men ≥ 102; women ≥ 88)	Trained personnel	UPF contributed 22.7% of total E intake. Individuals in highest quartile UPF intake had significantly higher BMI (0.80 kg/m^2^; 95% CI 0.53, 1.07), WC (1.71 cm; 95% CI 1.02, 2.40), and higher odds of being overweight (OR = 1.31; 95% CI 1.13, 1.51), obese (OR = 1.41, 95% CI 1.18, 1.69), increased WC (OR = 1.31, 95% CI 0.96, 1.32), and significantly increased WC (OR = 1.41; 95% CI 1.20, 1.66), compared with individuals in the lowest quartile.
Da Silveira 2017 [63]	Cross-sectional (2015) Brazil	Vegetarians > 16 years (*n* = 503)	Individual	UPF intake frequency (≥3 times per day) [DGB.2014] [28]	FFQ (number of items not specified)	BMI classified in overweight BMI ≥ 25 (16–59 years), BMI ≥ 27 (≥60 years)	Self-report #	Higher intake of UPF (≥3 times/day) was independently associated with overweight (OR = 2.33; 95% CI 1.36, 4.03).
Ali 2020 [64]	Cross-sectional (2018) Malaysia	Adults 18–59 years (*n* = 167) University personnel	Individual	UPF % total E intake (+continuous) [NOVA. 2018] [30]	2-day 24 h recall	BMI % Body fat	Trained personnel	UPF contributed 23 % of total E intake. No significant findings between ultra-processed food consumption BMI, body fat percent (*p* = 0.954).
Mendonca 2016 [65]	Prospective Cohort (1999–2012) 8.9 years median follow-up Spain	Adults Mean 37.6 years (*n* = 8451)	Individual	UPF intake servings/day (quartiles) [NOVA.2016] [22]	136-item FFQ	BMI classified in overweight/obesity (BMI ≥ 25), obesity (BMI ≥ 30).	Self-report #	Participants in the highest quartile of UPF consumption were at a higher risk of developing overweight/obesity (HR = 1.26; 95% CI 1.10, 1.45) compared with those in the lowest quartile of consumption.
Canhada 2020 [66]	Prospective Cohort (2008–2010) 3.8 years median follow-up Brazil	Adults 35–74 years (*n* = 11,827)	Individual	UPF % total E intake (quartiles) [NOVA 2016] [22]	114-item FFQ	Large weight gain (≥1·68 kg/year) Large WC gain (≥2·42 cm/year) Overweight/obesity (BMI ≥ 25 kg/m^2^) Obesity (BMI ≥ 30)	Trained personnel	UPF contributed 24.6% of total E intake. Participants in the highest quartile of UPF intake had greater risk of large weight (RR = 1.27; 95% CI 1.07, 1.50) and waist gains (RR = 1.33; 95% CI 1.12, 1.58), and of developing overweight/obesity (RR = 1.20; 95% CI 1.03, 1.40) compared with individuals in the lowest quartile.
Hall et al. 2019 [67]	Randomised Controlled Trial (2018, 4 weeks) USA	Weight stable adults Mean 31.2 years (*n* = 20)	Individual	Whole diet UPF vs. MPF diet (ad libitum) [NOVA.2018] [30]	Diets designed and analysed using ProNutra software	Energy Intake (kcal) Change in body weight (kg)	Trained personnel	Energy intake was greater during exposure to the UPF diet (508 ± 106 kcal/day; *p* = 0.0001). Participants gained 0.9 ± 0.3 kg (*p* = 0.009) during the UPF diet, and lost 0.9 ± 0.3 kg (*p* = 0.007) during the MPF diet.
**Cardio-metabolic risks**								
Lavigne- Robichaud 2017 [68]	Cross-sectional (2005–2009) Canada	Adults ≥ 18 years (*n* = 811)	Individual	UPF total E % intake (quintiles) [NOVA.2010] [25]	1 × 24-h food ** recall	Metabolic syndrome (MetS) (≥3 factor: high WC, HT TAG, BG; low HDL-C)	Trained personnel	UPF contributed 51.9% of total E intake. Those in highest quintile of UPF intake significantly associated with higher prevalence of MetS (OR = 1.90; 95% CI 1.14), higher prevalence of reduced HDL-C (OR = 2.05; 95% CI 1.25, 3.38), elevated fasting plasma glucose (OR = 1.76, 95% CI 1.04, 2.97) compared with those in the lowest quintile.
Nasreddine 2018 [69]	Cross-sectional (2014) Lebanon	Adults ≥18 years (*n* = 302)	Individual	UPF ‘pattern’ vs. MPF and PF ‘pattern’ (quartiles) [NOVA.2012] [54]	88-item FFQ	Metabolic syndrome (≥3 factors: high WC, HT, TAG, BG; low HDL-C)	Trained personnel	UPF vs. MPF were 36.5% vs. 27.1% of total E intake. Those in highest quartile MPF/PF significantly lower odds MetS (OR = 0.18, 95% CI 0.04, 0.77); hyperglycaemia (OR = 0.25, 95% CI 0.07, 0.98), low HDL-C (OR = 0.17, 95% CI 0.05, 0.60) compared with those in the lowest quartile. No significant association between MetS and UPF.
Lopes 2019 [70]	Cross-sectional (2008–2010) Brazil	Adults 35–74 years (*n* = 8468)	Individual	UPF % total E intake (terciles) [NOVA 2016] [22]	114–item FFQ	C-reactive protein (CRP) level (mg/L)	Trained personnel	UPF contributed to 20% total E intake. Women in highest tercile UPF intake had higher levels of CRP (arithmetic mean = 1.14; 95% CI: 1.04–1.24) than lowest tercile of intake, no significance when controlling for BMI. No significant association was observed in men.
Martinez Steele 2019 [71]	Cross-sectional (2009–2014) US	Adults ≥ 20 years (*n* = 6385)	Individual (National sample)	UPF Total E % intake (quintiles and continuous) [NOVA.2018.2019] [21,30]	2 available ×24-h recall, or 1 day otherwise.	Metabolic syndrome (≥3 factor of high WC, HT, TAG, BG; low HDL)	Trained personnel	UPF contributed 55.5% of total E intake. The highest quintile of UPF consumption was associated with higher MetS prevalence (PR = 1.28; 95% CI 1.09, 1.50) compared with the lowest quintile of UPF consumption. Each 10% increase in the consumption of UPF was associated with 4% increase in MetS prevalence (PR = 1.04; 95% CI 1.02, 1.07)
Mendonca 2017 [72]	Prospective Cohort (1999–2013) 9.1 years median follow-up Spain	Adult graduates (*n* = 14,790)	Individual	UPF E intake servings per day (tertiles) [NOVA.2016] [22]	136-item FFQ	Hypertension (BP: Systolic ≥ 140 mm Hg and/or Diastolic ≥ 90 mm Hg)	Self-report ξ	Participants in the highest tertile of UPF intake had higher risk of developing hypertension (HR = 1.21; 95% CI 1.06–1.37) compared with those in the lowest tertile of intake.

Results are presented for adjusted associations for potential confounders and statistically significant associations. NOVA refers to the food classification system [21] or earlier versions, as referenced; * Includes studies on all ages; ** includes beverages; # anthropometrics; ξ reported medical diagnosis, medication, or BP readings, ‡ results for adolescents are presented in Table 3; UPF: ultra-processed food (includes foods and beverages); BOA: Board of Agriculture; BMI: Body Mass Index [weight (kilograms)/height (metres)^2^]; E: energy in kilocalories or kilojoules; WHO: World Health Organisation; OR: odds ratio; CI: confidence interval; WC: waist circumference (cm); increased WC: (men ≥ 94; women ≥ 80; significantly increased WC (men ≥ 102; women ≥ 88); AO: abdominal obesity (men ≥ 102 cm; women ≥ 88 cm); FFQ: food frequency questionnaire; DGB: Dietary Guidelines for the Brazilian Population; HR: hazards ratio; RR: relative risk; MPF: unprocessed or minimally processed food; MetS: metabolic syndrome; HT: hypertension; TAG: triacylglycerol; BG: blood glucose; HDL-C: high density lipoprotein cholesterol; MPF and PF ‘pattern’: factor derived ‘pattern’ of mainly MPF and processed food (PF); CRP = C-reactive protein; BP = blood pressure.

**Table 3:** Overweight, obesity and cardio-metabolic risks as outcome (children and adolescents).

Study Details			UPF Exposure			Outcomes		Results
Publication Author(s) Year	Study Type (Year) Setting	Population (Number)	Extraction Level	Relative exposure [UPF Reference Year]	Data Collection Method	Health Outcome (Study Definition)	Data Collection Method	Key Findings
**Overweight and obesity**								
Louzada * 2015 [56]	Cross-sectional (2008-2009) Brazil	Children 10 to 19 years (*n* = 7534)	Individua (National Sample)	UPF % total E intake (quintiles) [NOVA.2012] [54]	2 × 24-h food ** intake record	WHO BMI-for-age Z-scores, in excess weight and obesity.	Trained personnel	UPF contribution ranged from ≤17% in lowest quintile to ≥52% in highest quintile. No significant association of UPF intake with mean BMI, excess weight or obesity was found.
Enes 2019 [73]	Cross-sectional (2016) Brazil	Adolescents 10–18 years (*n* = 200)	Individual	UPF % total E intake (quartiles) [NOVA.2018] [30]	58-items FFQ	Overweight Obesity BMI-for-age Z-scores	Trained personnel	UPF contributed 50.6% of total E intake. No association with UPF and anthropometric indicators.
Cunha et al. 2018 [74]	Prospective Cohort (2010–2012), 3 years median follow-up Brazil	Adolescents 15.7 years baseline, 17.6 years follow-up (*n* = 1035)	Individual	UPF intake (times/day) and daily E (kcal/day) [NOVA.2010] [25]	72-item FFQ	Trajectories of BMI (kg/m^2^)% body fat	Trained personnel	Baseline UPF intake was 9.7–12.5 times/day (boys) and 10.9–13.1 times/day (girls). There was no significant difference in BMI and % body fat trajectories during follow-up.
**Cardio-metabolic risk**								
Tavares 2012 [75]	Cross-sectional (2006-2007) Brazil	Adolescents 12–19 years (*n* = 210)	Individual	UPF E intake (kJ) (quartiles)[NOVA.2009] [9]	90-item FFQ	Metabolic syndrome (MetS) (≥3 factor of high WC, HT, TAG, BG; low HDL)	Trained personnel (assumed)	Highest intake of UPF (>3rd quartile) was associated with higher MetS prevalence (PR = 2.49; *p* = 0.012) than the lowest consumption.
Melo 2017 [76]	Cross-sectional (2012) Brazil	Adolescents 14–19 years (*n* = 249)	Individual	UPF intake frequency (<3 per week vs. ≥3 per week) [DGB.2014] [28]	84- item FFQ	Excess weight (BMI-for-age) High waist circumference High blood pressure	Trained personnel	UPF intake was ≥ 3 × week in 46.2% of adolescents. MPF intake inversely associated with excess weight. UPF intake was not significantly associated with excess weight, high WC and high blood pressure.
Rauber 2015 [77]	Prospective cohort (2001–2006) Brazil	Children 3–4 years at baseline; 7–8 years at follow-up (*n* = 345)	Individual	UPF % total E intake [NOVA.2014] [29,54]	2 × 24-h recall	Changes in lipid concentrations	Trained personnel	UPF contributed 42.6% at pre-school, 49.2% at school age of % E intake. For every 1% increase E intake from UPF, total cholesterol increased 0.43 mg/dL (*p* = 0.046), and LDL-C increased 0.369 mg/dL (*p* = 0.047) from age 3–4 to 7–8 years.
Costa 2019 [78]	Prospective Cohort (2001–2006), median follow-up age 4 to 8 years. Brazil	Children 4 years at baseline; 8 years at follow-up (*n* = 307)	Individual	UPF % total E intake [NOVA.2018] [30]	2 × 24-h recalls	Changes in BMI (kg/m^2^), Waist circumference (cm) Glucose profile and insulin resistance	Trained personnel	UPF contributed 41.8% preschool, 47.8% at school age of % E intake. Consumption of UPF consumption at age 4 was associated with increased delta waist circumference (B = 0.07 cm; 95% CI 0.01, 0.013) from age 4 to 8 years. No significant associations were observed for BMI, glucose profile and insulin resistance.
Leffa 2020 [79]	Prospective cohort (2011–2015) Brazil	Children 3 years at baseline; 6 years at follow-up (*n* = 308)	Individual	UPF % total E intake [NOVA.2018.2019] [21,30]	2 × 24-h recalls	Total cholesterol (TC) TAG	Trained personnel	UPF contributed 43.4% age 3 years, and 47.7% at age 6 years of % E intake. Those children in the highest tertile of consumption of UPF at age 3 had higher levels of TC (B = 0.22 mmol/L; 95 CI 0.04, 0.39) and TAG (B = 0.11 mmol/L; 95% CI 0.01, 0.20) at age 6 than those in the lowest tertile.

Results are presented for adjusted associations for potential confounders and statistically significant associations. NOVA refers to the food classification system [21] or earlier versions, as referenced. * Also included in Table 2; ** food includes food and beverages; UPF: ultra-processed food (includes food and beverages); E: energy in kilocalories or kilojoules; WHO: World Health Organisation; BMI; Body Mass Index; FFQ; food frequency questionnaire; MetS; metabolic syndrome; WC: waist circumference; HT: hypertension; TAG: triacylglycerol; BG: blood glucose; HDL: high density lipoprotein; PR: prevalence; LDL: low-density lipoprotein; DGB: Dietary Guidelines for the Brazilian Population; TC: total cholesterol.

## 3. Results

### 3.1. Overveiew of Identified Studies

Since the NOVA thesis was first published in 2009 [9] there have been 43 peer-reviewed studies reporting on UPF exposure and health outcomes that met this review’s eligibility criteria. The first study was published in 2012 [75] and 34 (79%) have been published since 2018. Studies were on adults (*n* = 31) (six excluded elderly, two excluded adults < 45 years), children ages 3–11 (*n* = 4), adolescents ages 10–19 (*n* = 5), and mixed ages (*n* = 3). Study types were ecological (*n* = 3), cross-sectional (*n* = 19), prospective cohort (*n* = 19), case–control (*n* = 1); and one randomised controlled trial (RCT). Studies were conducted in Brazil (*n* = 16) [53,56,62,63,66,70,73,74,75,76,77,78,79,80,81,82], France (*n* = 8) [61,83,84,85,86,87,88,89], Spain (*n* = 6) [65,72,90,91,92,93], USA (*n* = 4) [59,67,71,94], Canada (*n* = 2) [57,68], UK (*n* = 2) [55,60], and one each in Sweden [36], Lebanon [69] and Malaysia [64]. There was one study on 19 European countries [52], and one global study [37].

There were various levels at which researchers extracted food data. In the ecological studies, one study extracted at the household level and one combined household with national agriculture availability data [36,52] and the third, a global study on 80 countries, used national per capita sales volumes of UPF [37]. The remaining studies extracted data at the individual level, except one cross-sectional study which extracted at the household level [53]. Collection methods included sales-data (*n* = 1), household purchase records (range 7 days to one month, one combined with agricultural data) (*n* = 3), food-frequency questionnaire (FFQ) (range 88-880 items, one combined with 24-h recall) (*n* = 17), 24hr recall (*n* = 9), food intake records (from choice up to >3000 food items) (*n* = 9), food diary (*n* = 2), diet history interview (*n* = 1); and a designed diet for the RCT (*n* = 1). Relative UPF exposure was presented by per capita availability of sales by volume (*n* = 1), % household energy purchased (*n* = 3), % individual energy intake (*n* = 18), daily energy intake (*n* = 2), frequency(times/day)(*n* = 5), servings/day (*n* = 3), % total grams (*n* = 8), UPF ‘score’ (*n* = 1), UPF ‘pattern’ versus MPF ‘pattern’ (*n* = 1); and complete diet comparison (mainly UPF diet versus mainly MPF diet) for the RCT (*n* = 1).

Health outcome data collection methods were from self-report questionnaires (on diagnoses, anthropometrics, medication use, or medical histories), measurement by trained personnel, diagnoses by medical practitioners, or extracted from national registries or statistical records.

### 3.2. Ultra-processed Food Consumption

In studies on adults reporting the proportion of total energy intake from UPF, Malaysia was lowest (23%) [64]. Higher levels of consumption were reported in Spain (24.4%) [92], Lebanon (27.1%) [69], Brazil (20–29.6%) [56,62,66,70], France (29.9–35.9%) [61,86,87], Canada (45.1–51.9%) [57,68], and UK (53–54.3%) [55,60]. The highest levels were reported in the USA (55.5–56.1%) [59,71]. In studies in adults reporting servings per day or frequency of UPF consumption, one study in USA reported a mean of 4 times a day [94], and one study in Spain reported 1.4–5.3 servings per day from the lowest to highest quartile of UPF intake [91]. In French studies reporting consumption by weight of food, UPF ranged from 14.4 to 18.7% of total [61,83,84,86,87].

In children and adolescents (Brazil only) the proportion of total energy intake from UPF was reported as 41.8–43.4% at ages 3–4 years, 47.7–49.2% at ages 6–8 years [77,78,79] and 50.6% in adolescents [73]. One study reported 46.2% of adolescents consumed UPF weekly (median ≥ 3 times per week) [76], and another study reported frequency intakes varied from 9.9 (private schools) to 14.5 (public schools) times per day [74].

### 3.3. Studies Using Overweight, Obesity and Cardio-metabolic Risks as Outcomes (Adults)

Table 2 reports the findings of studies that investigated associations of UPF exposure and overweight, obesity and cardio-metabolic risks in adults.

#### 3.3.1. Overweight, Obesity and Related Factors

We identified 16 studies on adults investigating UPF exposure and ‘overweight’ (BMI ≥ 25), ‘overweight (BMI.25–30)’, obesity or related factors as outcomes. One study included all ages (including infants) and two studies included children or adolescents. Twelve studies reported direct associations with adverse health outcomes, one study showed no association and three studies showed mixed results (i.e., associations were not observed for all measures investigated or did not reach statistical significance).

There were three ecological studies identified: Juul et al. [36] analysed agricultural national food data and sampled approximately 4000 households. They found trends in food energy availability had risen in Sweden from 1980 to 2010, alongside a 142% increase in UPF portion in the diet. The prevalence of overweight rose in men (from 35% to 56%) and women (26% to 39%), and obesity rose in men (4.5% to 11%) and women (5% to 10%), closely tracking an increased share in food energy purchases from UPF [36]. Monteiro et al. [52] analysed budget surveys across 19 European countries. They found each percentage point increase in national household total food energy availability from UPF from 1991 to 2008 was associated with a higher national prevalence of obesity of 0.25% [52]. Vendevijvere et al. [37] investigated Euromonitor annual sales in 80 countries and found that increases in UPF volume sales were directly associated with population-level BMI trajectories. In drinks, every standard deviation increase (51 kg/capita in 2002) saw mean population BMI increase by 0.195 kg/m^2^ for men (*p* < 0.01) and 0.072 kg/m^2^ for women (*p* < 0.003). In foods, every standard deviation increase (40 kg/capita in 2002) saw mean population BMI increased by 0.316 kg/m^2^ for men (*p* < 0.001) with no significant association for women [37].

There were six cross-sectional studies on nationally representative samples. In Brazil, Canella et al. [53] found that household energy availability of UPF (purchased food items converted to kcal/day) was directly associated with average BMI and prevalence of excess weight or obesity. As UPF consumption rose from Quartile 1 to Quartile 4, the prevalence of excess weight rose from 34.1% to 43.9%, and prevalence of obesity rose from 9.8% to 13.1% [53]. In a second study in Brazil, Louzada et al. [56] found those in the highest quintile by percent energy intake of UPF had higher BMI and higher odds of being obese or overweight than those with the lowest quintile intake. For overweight individuals, the association was not significant [56]. In Canada, Nardocci et al. [57] found individuals in the highest quintile UPF by percent energy intake had a greater risk of obesity and overweight (BMI 25–30) compared with the lowest quintile [57]. In US adults aged 20–64 years from National Health and Nutrition Examination Survey 2005–2014 (NHANES), Juul et al. [59] found individuals in the highest quintile UPF by percent energy intake had higher BMI and waist circumference, and higher odds of overweight, obesity and abdominal obesity, with associations more pronounced in women [59]. In a UK study of 2174 adults, Adams et al. [55] found no significant association between UPF by percent energy intake and overweight or obesity. PCI was associated with the lowest odds of overweight and obesity, and MPF-CPI was associated with lower odds of being overweight [55]. In a second study in the UK on 6143 adults aged 19–96 years, Rauber et al. [60] found individuals in the highest quartile UPF by percent energy intake had higher BMI and waist circumference, and a higher odds of having obesity than those in the lowest quartile [60].

There were four cross-sectional studies on non-nationally representative samples. In Brazil, Silva et al. [62] assessed 8977 active and retired civil servants aged 35–62. They reported individuals in the highest quartile of percent energy intake compared with lowest quartile had higher BMI and waist circumference, as well as higher odds of being overweight (BMI 25–30), being obese, having increased waist circumference, or having significantly increased waist circumference [62]. In France, Julia et al. [61] studied 74,470 participants in a web-based Nutri-Santé cohort. They measured UPF intake by percent weight by quartiles and found UPF was associated with overweight (BMI 25–30), and energy-weighted quartiles were associated with overweight (BMI 25–30) and obesity [61]. In smaller studies, De Silveira et al. [63] examined vegetarians over 16 years old (*n* = 503) and found overweight in high UPF consumers was 38.3% versus mean 23.5%. In Malaysia, Ali et al. [64] studied adults 18–59 years (*n* = 167) and found no association between UPF and BMI or percentage body fat.

There were two prospective cohort studies. In Spain, Mendonca et al. [65] followed 8451 middle-aged graduates not overweight at baseline for a median of 8.9 years. Participants in the highest quartile of UPF intake (servings/day) had a 26% higher risk of developing overweight relative to the lowest quartile [65]. Considering those lost to follow-up, and those with repeated exposure measurement at 10-year follow-up, there remained a 24% risk and 19% risk, respectively [95,96]. In Brazil, Canhada et al. [66] followed 11,827 adults for 3.8 years. Participants in the highest quartile of UPF by percent energy intake had a 27% greater risk of developing ‘large weight’ and 33% risk of ‘waist gains’ than those in the lowest quintile. In those not overweight at baseline, there was a 20% risk of developing overweight compared to the lowest quartile. In crude analysis, for those overweight and not obese (BMI 25–30) at baseline there was an increased risk of developing obesity, but this did not reach significance in the fully adjusted model considering baseline BMI [66].

There was one randomised controlled crossover trial. In 20 US weight-stable adults free of disease at baseline (mean age 31.2 ± 1.6 years; mean BMI 27 ± 1.5 kg/m^2^), Hall et al. [67] found that an a ultra-processed diet caused weight gain. Participants were provided two complete diets (either ultra-processed (81.3% energy from UPF) or unprocessed (0% UPF)) matched for calories, sugar, fat, sodium, fibre and macronutrients, in random order, in excess of daily energy requirements to consume ad libitum for 14 days each diet. Participants had a greater eating rate, consumed more energy (508 ± 106 kcal/day; *p* = 0.0001) and gained weight (0.9 ± 0.3 kg; *p* = 0.009) on the ultra-processed diet, and lost weight 0.9 ± 0.3 kg (*p* = 0.007) during the unprocessed diet. Weight changes were highly correlated with energy intake (*r* = 0.8, p < 0.0001) [67].

#### 3.3.2. Cardio-Metabolic Risks

We identified five studies assessing cardio-metabolic risk factors in adults. Three showed an association of UPF exposure and adverse health outcome, one study reported mixed results, and one study had no observed association. There were four cross-sectional studies. On a representative sample of US adults, Martinez Steele et al. [71] found the highest quintile of UPF intake by percent energy was associated with 28% higher metabolic syndrome prevalence rate compared to the lowest quintile intake. Each 10% increase in consumption was associated with a 4% increase in metabolic syndrome prevalence. The association was strongest in younger adults [71]. In a study in Brazil of 8468 adults, Lopes et al. [70] found women in the highest tercile of UPF by percent energy intake had higher levels of C-reactive protein (CRP) than those in the lowest tercile. When controlling for BMI, the association was not statistically significant. No association was found in men [70]. In Canada, Lavigne-Robichard et al.’s [68] study of adults (*n* = 811) found those in the highest quintile of UPF intake by percent energy had a higher prevalence of metabolic syndrome, reduced high-density lipoprotein cholesterol and elevated fasting glucose [68]. In a Lebanese study of adults (*n* = 302), Nasreddine et al. [69] found those in medium/high adherence to a factor-derived MPF and PF ‘pattern’ vs. low adherence had lower odds of metabolic syndrome, hyperglycaemia and low HDL-C. No significant association was observed with metabolic syndrome and UPF ‘pattern’ [69]. There was one prospective cohort study. In Spain, Mendonca et al. [72] followed 14,790 adult graduates for a median 9.1 years. Participants in the highest tertile of UPF consumption (servings/day) had a 21% higher risk of developing hypertension than those in the lowest tertile [72].

### 3.4. Overweight, Obesity and Cardio-Metabolic Risks as Outcomes (Children and Adolescents)

There were eight studies on children and adolescents assessing UPF exposure and overweight, obesity body weight or cardio-metabolic risks as an outcome, which are presented in Table 3. Three studies showed associations of UPF intake and outcomes, four showed no associations and one had mixed results. All studies were from Brazil.

There were three studies on overweight and obesity. In a nationally representative cross-sectional sample on children and adolescents aged 10–19, Louzada et al. [56] found no significant association with UPF and mean BMI, overweight/obesity or obesity. In a small cross-sectional study on adolescents aged 10–18 years (*n* = 200), Enes et al. [73] found no association between UPF consumption and anthropometric indicators. In a prospective cohort study, Cunha et al. [74] followed 1035 adolescents (mean age 16 years) for three consecutive years and found no significant difference in BMI and percentage body fat trajectories.

There were five studies assessing cardio-metabolic risks. In a cross-sectional study on adolescents aged 12–19 years (n = 210), Tavares et al. [75] showed those with high UPF consumption (>Quartile 3) was associated with higher metabolic syndrome than those in the lowest quartile. In a second cross-sectional study on adolescents aged 14–19 years (n = 249), Melo et al. [76] found that while UPF was not associated with excess weight, hypertension or high waist circumference, consumption of MPF was inversely associated with excess weight. There were three prospective cohort studies. Following children aged 4–8 years (*n* = 345), Rauber et al. [77] reported UPF consumption (by percent energy intake) at pre-school age was a predictor of higher total and LDL cholesterol at school age. In a follow-up of a previous RCT on 307 children aged 4–8 years, Costa et al. [78] found for every increase of 10% energy intake from UPF, delta waist circumference increased by 0.7 cm. Further, higher UPF consumption at pre-school age was a predictor of an increase in delta waist circumference from pre-school to school age. No association with fasting glucose or insulin was detected [78]. Leffer et al. [79] following young children, for three years found those children in the highest tertile of UPF energy intake at age 3 had higher levels of total cholesterol, and tri-acyl glycerol at age 6 than those in the lowest tertile [79].

### 3.5. Studies Using Diseases and Mortality as Outcomes

Table 4 reports the findings from studies that evaluated the association of UPF exposure and diseases and mortality as outcomes.

#### 3.5.1. Cancer

In a case–control study investigating breast cancer in Brazil, Quiroz et al. [81] matched 59 women with breast cancer to 59 non-cancer controls. They found regular consumption of UPF (>5 days/week) was identified as having 2.35 times higher odds of breast cancer [81]. In a longitudinal study, Fiolet et al. [84] found a higher incident rate of cancer with UPF exposure. For every 10% increment in the proportion of UPF in the diet (by percentage grams), there was a 12% higher risk for total cancers, and 11% increased risk for breast cancer [84].

#### 3.5.2. Cardiovascular Disease

In a prospective cohort study on 105,109 adults over a median period of 5.2 years, Srour et al. [83] found a higher incident rate of all cardiovascular disease (CVD) in those with the highest intake of UPF consumption (by percentage weight). Those in the highest quartile intake had a 12%, 13% and 11% increased risk of all CVD, coronary heart disease and cerebrovascular disease, respectively [83].

#### 3.5.3. Type 2 Diabetes

In France, Srour et al. [87] followed 104,707 participants free of type 1 or type 2 diabetes for a median 6.0 years. Participants with a higher proportional intake (by weight) of UPF in the diet had significantly higher risk of type 2 diabetes. A 10% increase in UPF in the diet was associated with a 15% higher risk of type 2 diabetes [87].

#### 3.5.4. Mortality

In a mortality study of US adults aged over 20 years over a median follow-up of 19 years, Kim et al. [94] found those participants with the highest quartile of consumption of UPF (frequency/day) had a 31% higher risk of all-cause mortality. There was no observed association with CVD mortality [94]. In a study of 44,551 individuals over a median period of 7.1 years in France, Schnabel et al. [86] found that participants who consumed a higher proportion of UPF (by proportion of weight in grams) had a higher risk of all-cause mortality. Each 10% increment in proportion consumed was associated with a 14% higher risk of all-cause mortality [86]. In a prospective cohort study in Spain of 19,899 individuals, Rico-Campa et al. [91] found participants in the highest quartile of UPF consumption (by servings per day) had a 62% higher risk of all-cause mortality. For each additional serve, there was an 18% higher risk. No significant associations were found with CVD and cancer mortality [91]. In a second prospective cohort study in Spain, Blanco-Rojo et al. [92] followed 11,898 individuals for 7.7 median years. They found participants in the highest quartile of consumption (by percent energy) of UPF had a 44% higher risk of all-cause mortality. Iso-caloric substitution of UPF with MPF was associated with a decrease in mortality [92]. 

### 3.6. Studies Using Other Disorders and Conditions as Outcomes

Table 5 reports the findings of studies that investigated associations of UPF exposure and other disorders and conditions as outcomes.

#### 3.6.1. Gastrointestinal disorders

In a cross-sectional study in France of 33,343 adults, Schabel et al. [85] found participants in the highest quartile of UPF intake by percentage share (g/day) had a higher risk of irritable bowel syndrome (IBS)—either alone or when considering IBS and functional dyspepsia (FDy)—compared to those in the lowest quartile. There was no association of UPF intake with FDy alone [85]. In a prospective cohort study in France, Vasseur et al. [88] found no significant association with UPF intake and inflammatory bowel disease.

#### 3.6.2. Depression

In a prospective cohort study in France, Adjiade et al. [89] followed 26,730 individuals without depressive symptoms at baseline by Centre for Epidemiological Studies Depression Scale (CES-D) scale) for mean 5.4 years. Participants in the highest quartile of UPF intake by percentage share (g/day) had a 31% increased risk of developing depression than those in the lowest quartile. An estimated 10% increase in UPF consumption was associated with a 21% increased risk of depression [89]. In Spain, Gomez-Donoso et al. [90] followed 14,907 university graduates free of depression (by diagnosis history) for a median 10.3 years. Participants in the highest quartile intake of UPF (percentage share g/day, energy-adjusted) had a 33% higher risk of developing depression than participants in the lowest quartile [90].

#### 3.6.3. Frailty

In a prospective cohort study in Spain, Sandoval-Insausti et al. [93] followed 1822 adults >60 years free of frailty for 3.5 years. Those in the highest quartile of UPF intake (percent total energy) had 3.67 times higher odds of frailty than those in the lowest quartile [93].

#### 3.6.4. Asthma

In a nationally representative sample of students aged 13–16 years, Melo et al. [80] found an association between a higher UPF score (based on consumption of selected sub-categories of UPF) and asthma in a dose-response manner. In a prospective cohort study in Brazil, Azerado et al. [82] followed 2190 children aged 6–11 years and found no association between UPF consumption (percent energy intake) and wheeze, asthma or severe asthma.

### 3.7. Quality Appraisal

The limitations and strengths found in the appraisal were used to support the critiques of studies in the discussion section. Further details are described in Supplementary Material Table S3.

## 4. Discussion

This review identified 43 studies investigating associations between UPF exposure and various health outcomes and related risks, with most studies reporting more than one outcome. In 37 studies, there was at least one statistically significant association between UPF exposure and at least one adverse health outcome. No study reported an association between UPF exposure and beneficial health outcomes that reached statistical significance, were adjusted for covariates and―in prospective studies―were reported at follow-up. Beneficial outcomes were found associated with diets higher in MPF. The findings can be summarised and classified as follows: (1) in 21 studies assessing overweight, obesity and cardio-metabolic risks in adults, 15 reported significant associations and adverse health outcomes, four reported mixed associations (that is some adverse health outcomes, and some with no associations), and two found no significant associations; (2) in eight studies assessing disease or mortality, five found significant associations and adverse health outcomes, and three found mixed associations; (3) in seven studies investigating other disorders and conditions, five found only significant associations and adverse health outcomes, and two found no associations; (4) in eight studies reporting on overweight, obesity and cardio-metabolic risks in children and adolescents, three found significant associations with adverse health outcomes, two found mixed associations and three found no associations. Seven studies in adults found associations between diets high in MPF (or MPF combined with PCI or PF) and beneficial health outcomes. A study on mortality found iso-caloric substitution of UPF with MPF was associated with a decrease in mortality.

Exposure to UPF was found to be associated with population BMI trajectories, overweight and obesity prevalence as well as individual BMI, overweight including obesity (BMI ≥ 25), overweight excluding obesity (BMI 25–30), obesity, weight gain, abdominal obesity, metabolic syndrome, high blood pressure, blood lipids, blood glucose, type 2 diabetes, cardiovascular disease, coronary artery disease, cerebrovascular disease, overall cancers, breast cancer, frailty, IBS, depression and mortality (in adults), and asthma, blood lipids, metabolic syndrome and delta waist circumference (in children or adolescents). The studies that found no associations were assessing BMI, overweight, obesity, metabolic syndrome, inflammatory bowel disease, CRP (men), prostate and colorectal cancer, and mortality in CVD and cancer (in adults) or anthropometric indicators, high blood pressure, blood glucose and insulin resistance (in children). Although they showed no associations with UPF and investigated outcomes, two studies on adults and one on adolescents demonstrated that a higher intake of MPF-PCI had lower odds of overweight/obesity [55], a higher intake of MPF/PF ‘pattern’ had lower odds of metabolic syndrome [69], and MPF intake was inversely associated with overweight [76]. Three of the French studies found an association between diets higher in MPF consumption and lower risk of adverse health outcomes [83,84,87]. The RCT found participants lost weight and had improved bio-markers during the MPF diet phase [67].

### 4.1. Overweight, Obesity and Cardio-Metabolic Risks as Outcomes (Adults)

The majority of studies investigating UPF exposure and adverse health outcomes of overweight, obesity or cardio-metabolic risks showed associations. The WHO defines overweight and obesity as ‘abnormal or excessive fat accumulation that may impair health’ [48] and states that weight gain, at whatever the original weight, is a risk for disease [46,47]. Overweight and obesity are both significant risks for CVD, cancers and mortality, with body fat distribution, blood lipids, hypertension and blood glucose related risks [97]. Studies in individuals that measured only mean BMI, overweight and obesity [53,56,57,59,61,66] are indicative of risk. However, studies including body fat anthropometrics, abdominal obesity or biomarkers [59,60,62,67], the two prospective studies and RCT showing weight gain over time [65,66,67], the prospective study on hypertension [72], a study on metabolic syndrome on a nationally-presentative sample [71], and a smaller study [68] provide stronger support of the risk of UPF exposure. Moreover, the ecological studies tracing UPF purchase country comparisons [52] or time-trend analyses within [36] and between [37] countries, all showed an association with or trajectories of higher exposure to UPF and the risk to population prevalence rates or weight trajectories over time within or between countries.

The ecological studies’ strengths were the use of national obesity statistics [36,52], large standardised datasets [37] and nationally representative population surveys with complex sampling [52], which support an association of UPF availability and rising rates of global obesity and population mean BMIs. The strengths of the cross-sectional studies were in nationally representative samples (*n* = 6) or large samples (*n* = 2) with data extraction at individual intake, except one by household purchases [53] and outcome measurements by trained personnel in all studies except two [61,63] and partially in a third [57]. Critically, in two prospective cohort studies, Canhada et al. [66] and Mendonca et al. [65] demonstrated individuals not overweight or obese at baseline moved over time to BMI parameters of significant risk, with individuals in the highest quartile of UPF having the highest risk. The studies’ strengths were demonstrating both the risk over time for an individual, and group comparison of higher to lower UPF exposure. The Mendonca et al. study was on younger health professionals (mean age 37.6), a group who may be more motivated to follow a healthy diet pattern [65]. Results may not be applicable to the general population and in fact may be worse in those less motivated to follow a ‘healthy’ diet, which would make the association of UPF exposure and body weight gain even stronger. An interventional RCT study by Hall et al. [67] demonstrated weight gain on consumption of UPF diet and weight loss on an unprocessed diet. While whole diets are unable to be blinded, participants were blinded to glucose readings, and to weight change by wearing loose-fitting clothing.

Ecological studies were limited by methodologies unable to separate age and gender [36,52], the exclusion of food purchases outside the home or take-away food outlets [37,52], or time lags between the collection of survey data and obesity data of up to five years [52]. Ecological studies are considered a relatively weak study design due to the risk of bias in interpreting results that are observations at the population level, not necessarily translatable to individuals; and all cross-sectional studies are unable to determine causality and temporality and have the potential for confounders. In studies with mixed results, one study showed that in women (but not men), there was an association of UPF exposure with higher CRP, although not after controlling for BMI [70]. The authors posited that adiposity was driving the inflammation; however, it is also possible that inflammation could be driving adiposity [98,99] and that diet-induced inflammation could facilitate further pro-inflammatory events [98,100]. Three studies did not find any association of UPF exposure and overweight, obesity or cardio-metabolic risks. A Malaysian study on university personnel found no association with BMI or percentage body fat [64]. The study was limited by its small sample size and noted difficulties in the application of NOVA to the national food database. One study on a nationally representative sample in the UK showed no associations of UPF and body weight parameters [55]; whereas, the consumption of MPF-PCI pattern had lower odds of overweight and obesity. Likewise, in a study that showed no significant association with UPF and metabolic syndrome [69], there were lower odds of metabolic syndrome, low HDL and hyperglycaemia with higher MPF/PF intake. Both studies provide support for MPF diets over UPF. The UK study used an early NOVA classification and a more recent study found direct associations between UPF exposure and obesity [60].

### 4.2. Overweight, Obesity Cardio-Metabolic Risk as Outcomes (Children and Adolescents)

In investigations of cardio-metabolic risks in children and adolescents, three prospective studies following young children from ages three (or four) through to ages six (or eight) years showed a higher intake of UPF at age three (or four) predicted higher total cholesterol, LDL cholesterol, tri-acyl glycerol and/or increased delta waist circumference at age six (or eight) [77,78,79]. In cross-sectional studies, one study on adolescents showed an association of higher UPF intake and metabolic syndrome [75], and a second on adolescents showed no association with overweight, higher waist circumference and high blood pressure [76]. Three studies investigating overweight and obesity on older children and adolescents aged 10–19 years showed no association of UPF with anthropometrics measured [56,73,74]. Direct comparisons of the studies were difficult due to differing study designs, exposure measurements and outcomes and methodological weaknesses. Two of the studies reported quite high mean energy intakes of >4000 Kcal/day (>17,000 KJ) [73,74], and one study had a very small sample (*n* = 200) with a low power of the statistical test [73]. In the prospective study there was a large drop-out rate of 43% [74]. The authors reported cross-sectional results and prospective changes were not statistically significant.

Children and adolescents have age-appropriate energy and nutrients needs for normal growth, development, cognitive advancement, physical activity and prevention of micro-nutrient deficiencies [101], in addition to consideration of risk for NCDs. The three studies on young children consistently showed a higher proportion of UPF were consumed at school age compared to preschool age and consistently showed direct associations between UPF consumption and cardio-metabolic or obesity risk. These provide strong support for an association between UPF consumption and cardio-metabolic risks in young children, and there is a concern that UPF consumption rises with age in childhood. The less strong associations in studies in older children and adolescents generally may be attributed to the role of the growth spurt in the pubertal years together with higher physical activity limiting excess weight. Nevertheless, dietary habits set in childhood and adolescence may be difficult to change, and there is a need to study post-pubertal young adults in future studies. The age bracket from post pre-school to pre-adolescence (~age 7–10) was under-represented in the studies reviewed. With the studies’ methodological differences and limitations, it is difficult to draw any firm conclusions in older children and adolescents.

### 4.3. Diseases and Mortality as Outcomes

There were four studies investigating UPF exposure and disease, and four studies investigating UPF and mortality. All of the studies showed a direct association with UPF intake and outcomes of breast cancer, total cancers, cardiovascular disease, coronary heart disease, cerebrovascular disease and mortality. There were no associations shown for prostate and colorectal cancer, cancer mortality and CVD mortality. Seven of the studies were prospective cohort studies and there was one case–control study in breast cancer cases compared to matched controls. Health outcome measurements were by validated scales of measure, medical records, confirmed diagnosed history and (for mortality) national death registries. All studies reported statistical analyses adjusting for appropriate covariates.

### 4.4. Studies that Investigated Other Disorders and Conditions as Outcomes

Seven studies investigated exposure to UPF and other disorders. Direct associations were observed with UPF intake and IBS, depression and frailty in adults [85,89,90,93]. Frailty, in turn, is a risk for stressors and less inclination for physical activity, and is in itself a risk for CVD [102]. One prospective study showed no association with inflammatory bowel disease. In the two studies investigating asthma, a prospective cohort study in children aged 6–11 years showed no association [82], and a cross-sectional study drawn from a representative sample of adolescents showed a direct association [80]. The studies are not directly comparable due to different study designs, age brackets and UPF definition, including 18 and 21 UPF groups in one study, and six in the other. While disorders and conditions are not considered main contributors to the global burden of death, they are main contributors to the burden of non-fatal illnesses and DALYs lost [11]. It is important to recognise the contribution diet—and in particular, UPF—is having on the reported incidence rates of these conditions.

### 4.5. Dietary Assessment, UPF Definitions and Confounding Variables

Most of the studies found UPF exposure was associated with one or more adverse health outcome. However, the differences in studies’ methodologies make comparisons between studies difficult.

Firstly, concerning the UPF definition, the original NOVA three-group (now four-group) food processing classification was first proposed in 2010 [9,25]. This was followed by development [26,27,29,54] and insertion into the dietary guidelines for the Brazilian population in 2014 [28]. An update occurred in 2016 [22], and further explanatory documents were published in 2018 and 2019 [21,30]. NOVA history has been described elsewhere [5,22,29]. The studies described in this review used these varying historical versions and additional studies on NOVA [33,58] as their reference for UPF definition, resulting in some differences in the foods classed as UPFs. Further details are provided in Supplementary Material Table S2. For example, the studies using early definitions with only three food groups (‘ultra-processed food products’ contained the current groups three and four; that is, with cheese and all bread regarded as UPF and pasta regarded as PCI) [53,55,56,69,74,75,81], and artisanal bread included as UPF until 2014 [36,59,77], may not directly compare to studies using later definitions. Studies also ranged in the reporting of their application of NOVA from the provision of only a reference and brief description [70,75] or short food lists mismatched to reference [68] to detailed supplementary lists and/or explanations of analysis of national food databases of >3000 foods [61].

Secondly, against a broad range in the strengths and limitations of various data extraction methods [103], authors noted additional benefits or constraints of instruments in application to NOVA, and to national food databases. Food diaries (able to account for seasonal or weekly variations) [60] and 24-h recalls [59,78] record actual foods consumed and preparation methods, with fewer difficulties were noted in application than reported in the use of FFQs. FFQs included between 55 and 880 items and UPF ranged from 6 to >21 sub-groups, with application to NOVA constrained to foods listed. In several instances, foods were classified differently across surveys due to different food systems in country-specific contexts. For example, some FFQs do not separate the bread type and in the US and UK it is mainly UPF [60,104], whereas in France and Spain it is mainly artisanal [52,61]. Additional complexities included application to food databases with a lack of information on the differentiation of canned food into PF or UPF, databases disaggregating foods to nutrient content rather than processing type (e.g., frozen pizza disaggregated to component ingredients could inadvertently be classed as PF or PCI, and not correctly as UPF), and disaggregating handmade dishes (e.g., Bolognese pasta) into major food-items in the recipe (e.g., group 1, pasta) rather than underlying ingredients (pasta, meat, sauce, oil), which is the recommended approach [59,105].

Thirdly, most studies used percent energy intake as a measure of relative exposure of UPF, which is difficult to compare to studies that used frequencies (times per day), servings per day, or consumption of an UPF ‘pattern’. The French NutriNet-Santé studies used percentage weight per day, arguing the need to account for UPF that have no or less energy value (e.g., artificially sweetened beverages) than regular alternatives [61,83,84,85,86,87,88]. Ideally, prospective studies collect food data more than once, yet some studies provided no details or collected data only once [72]. Conversely, the NutriNet-Santé studies collected data by the use of three 24-h records every six months for two years to establish a baseline diet and UPF intake [61,83,84,85,86,87,88].

Lastly, adjustments for confounding variables differed among the studies. In particular, one ecological study did not have access to data on confounders [36]. Some studies did not adjust for total energy, which confounds the risk for weight gain which is in itself a risk for CVD, cancer and type 2 diabetes; or for physical activity [55,63,76] (or used proxies [56]), which confounds the risk for CVD. These factors make comparisons between studies difficult.

### 4.6. Consistency with Findings in Other Reviews and Studies

That UPF exposure has been found to be associated with numerous diseases and conditions across a broad range of ages, populations and settings, underlines the role of diet quality as assessed by the ‘extent and purpose of industrial processing’ (as defined by NOVA [21]) influencing human health. This finding is consistent with findings reported in other reviews [2,3,4,5,7]. An early narrative review [4] and a recent systematic review [5] investigating studies on UPF exposure and obesity and/or cardio-metabolic risks, and a systematic review investigating body fat in childhood [2], similarly found most studies showed direct associations. The recent review [5] and a systematic review of studies of children’s dietary intake in relation to Brazil’s Dietary Guidelines [106] also noted that the literature was marred by inconsistencies in study definitions and methods. However, our review found the more recent studies—particularly the French, Spanish, US and UK studies since 2018—which do not have the NOVA classification limitations as in earlier studies, have adjusted for confounders, and expert teams have evolved comprehensive processes for analysis and classification of national surveys and food-databases in the application of NOVA classification [37,59,60,61,65,67,71,72,83,84,85,86,87,88,89,90,91,92,93,94]. Our review found similar UPF consumption trends as previously reported of up to >50% in high income countries and up to 30% in middle income countries. Notably Spain (24.4%) and France (29.9–35.9%) had lower UPF intake than Canada, UK and the USA (45.1–56.1). In Brazil, children (41.4–49.2%) and adolescents (50.6%) had higher consumption rates than adults (20–29.6%). In our review, one prospective study in adolescents showed an association of UPF exposure and beneficial effects at baseline that did not reach statistical significance at follow-up [74]. In a book chapter reporting a study in Kenya, beneficial nutritional effects were found associated with UPF in children [107]. Food data, however, were extracted at the household level and estimated to individuals. 

### 4.7. Proposed Mechanism of Effects

Evidence for the mechanisms underpinning the link between UPFs and adverse health outcomes is still emerging. Proposed mechanisms include a poor nutritional profile (i.e., UPFs are vectors for added sugars, sodium and trans-fats) and displacement of MPFs in the diet [33,34,35,58,108,109,110,111,112,113,114], higher glycaemic load and reduced gut–brain satiety signalling resulting from altered physical properties created by the processing of foods [115,116,117,118], carcinogens formed during high-temperature cooking (e.g., carbohydrate-rich foods with acrylamide) [119,120], and inflammatory responses linked with acellular nutrients and industrial food additives, gut microflora dysbiosis and increased intestinal permeability [98,121,122]. Certain properties of UPFs may promote overconsumption [123], including their often ubiquitous availability and convenience [124,125,126], palatability and quasi-addictiveness [127,128] and intensive marketing practices used to promote purchasing and consumption, especially among children and adolescents [129,130,131].

The NutriNet-Santé prospective studies investigating CVD and cancer found participants with high UPF exposure consumed diets higher in energy intake, sodium, lipids and carbohydrate, and participants in the CVD study had lower intakes of MPF, fruit, vegetables and fibre; in the study on type 2 diabetes, participants with higher UPF intake consumed diets higher in energy intake, sugar and sodium, and lower in wholegrain cereals, fruit, vegetables and fibre [83,84,87]. However, the studies reported associations of UPF exposure and the diseases remained even after adjusting for energy intake, salt, sugar/added sugar and Western dietary patterns (for CVD and cancer), and for a low ‘healthy’ diet pattern/diet quality (CVD and type 2 diabetes) or fibre (CVD). Those associations remained after adjustments, which suggest something other than nutritional factors may be contributing to the recorded observations. Moreover, in the CVD study, associations were observed for individual UPF food groups such as beverages, fats and sauces, meat, fish and eggs, sugary products and salty snacks; yet associations were stronger when the overall amount of UPF was considered rather than specific food groups. The authors postulated possible synergistic effects of many compounds in UPF. Similarly in other studies adjusting for overall diet ‘quality’, the associations between UPF exposure and adverse health outcomes or mortality remained [91,94].

In the RCT by Hall et al. [67], energy intake was greater during the UPF diet; the participants consumed more fat and carbohydrate, and gained weight and body fat. The diets were controlled for presented calories, energy density, macronutrients, sugar, sodium and fibre, providing causal evidence that something other than risk nutrients in UPFs causes energy intake and weight gain, and an increase in body fat with the consumption of a diet higher in UPFs. The study showed reduced secretion of the hunger hormone ghrelin, as well as increased levels of the satiety hormone PYY (peptide YY) with the unprocessed diet. Hence, relative to UPFs, unprocessed foods may stimulate more efficient regulation of biological mechanisms controlling hunger and satiety. The study also showed a reduction in the inflammatory bio-marker hs-CRP (highly sensitive CRP) with the unprocessed diet, and inflammation has been demonstrated to be coupled with satiety signals in animal studies [98].

Critics of NOVA have argued that ‘healthy dietary patterns’ are linked to nutrient intakes [132], and any adverse health effect seen in studies on UPF can be explained by nutritional factors [133] such as the presence of high levels of sugar, fat and salt. However, the literature indicates that the effects of ultra-processing on a food’s physical structure and chemical composition is independent of and therefore in addition to the effects of ‘risk’ or ‘positive’ nutrients present in the food [67,83]. Moreover, a defining characteristic of the NOVA classification is classifying added sugar, oils and fats, and salt as ingredients (Group 2). In UPF (Group 4), it is the high amount of these ingredients combined with processing techniques (deconstruction of the food matrix, removal of water) and addition of cosmetic additives, that explains the final characteristic of UPFs (high content of sugar, salt, fat; high energy density; and palatable) [21]. A further explanation of the mechanism by which UPFs influence health outcomes is that their inclusion in the diet displaces ‘protective’ MPFs, with low intakes of MPFs increasing the risk of CVD, cancer, type 2 diabetes and mortality [5,10].

### 4.8. Strength and Limitations

This is the first study to perform a systematic search for studies that have investigated UPF exposure and health outcomes, combined with a narrative review. The strengths of this review are the broad inclusion of study types, populations and health outcomes, a quality assurance process that involved a team working together and reaching consensus-based decisions, and undertaking a synthesis of the evidence to provide a coherent assessment of the role UPFs in influencing human health.

This review has limitations. We did not assess studies investigating participants with pre-existing conditions (including overweight or obesity) where UPF intake was considered an outcome. Interestingly such studies have similarly found direct associations of health conditions and UPF consumption [134,135]. The review was limited by the constraints within the studies under review which were mainly observational studies that cannot deduce cause and effect relationships. The smaller studies were difficult to compare to studies with national representative samples, yet were important representations of special groups or varying cultures [63,64,68,69].

### 4.9. Future Priorities for Nutrition Research and Policy Practice

With the rapidly growing number of studies reporting associations between UPFs and adverse health outcomes, and NCDs, there is an increasing need to take on board the contribution of these foods to the global burden of disease, as well as the need to act on multiple levels, including through government regulation, to improve the health prospects of current and future generations.

In the future, priority research activities to further develop the body of evidence for associations between UPF intake and health outcomes include continuing to analyse existing cohorts, conducting clinical trials, extending the cohort and clinical studies to a range of population groups, such as those in low- and middle-income countries, as well as childhood and adolescence years, to determine whether UPF influences on metabolism start before adulthood, and investigating plausible causal pathways to provide mechanistic understandings of the nature of the health associations. Further priorities include standardisation of application of the NOVA classification and analysed national databases, and a protocol for including confounder adjustment, especially weight parameters (with obesity being a known risk for CVD, cancer and type 2 diabetes) and low physical activity (a risk for CVD) [97].

With this solid evidence base the need for NOVA and UPF issues to be applied in policy practice, for example to food labelling and informing food procurement activities, is even more relevant. Food-based dietary guidelines and nutrition policy actions should consider incorporating the concept of ultra-processing to describe the healthiness of individual foods within the overall dietary pattern.

## 5. Conclusions

This review has shown that a high dietary intake of ultra-processed foods is associated with a range of adverse health outcomes, and non-communicable diseases, disorders and conditions, thereby bearing the potential to significantly influence the global burden of disease. Moreover, evidence suggests a higher risk of all-cause mortality with high consumption of ultra-processed foods. No study reported an association between UPF and beneficial health outcomes. The review has also shown beneficial outcomes were associated with diets higher in unprocessed and minimally processed foods. Although the majority of studies are of observational nature, the evidence base concerning plausible biological mechanisms supporting the observed associations is steadily evolving. The findings in this review support the notion that inferring health effects from individual nutrients and ingredients in foods is insufficient, and that industrial processing, and its extent and purpose, may add accuracy and reliability in predicting and explaining associations between foods and health outcomes. The considerable and growing body of evidence supporting the use of ultra-processed foods as a scientific concept to assess the ‘healthiness’ of foods within a dietary patterns context has the potential to improve future development of dietary guidelines as well as nutrition policy actions.

## Figures and Tables

**Table 1:** Inclusion and exclusion criteria used for screening studies.

	Description
Inclusion criteria	Stated aim was to evaluate the relationship between UPF exposure and a given health outcome or outcomes. Healthy, free-living human subjects over two years of age (except when included in studies on all ages), any country/ethnicity. UPF category or sub-categories used in the study were defined and referenced to the NOVA food classification system, including the Dietary Guidelines for the Brazilian Population, based on NOVA. Original empirical research articles published in peer-reviewed journal, with full text available. Had clearly stated aim(s) and objectives, well defined and appropriate method, a clear statement of results, and conclusions consistent with the study findings. Published between 1 January 2009 and 21 May 2020. Available in the English language.
Exclusion criteria	Study aim was to evaluate UPF exposure with non-health related outcome(s) or in non-human subjects or human populations with pre-existing health conditions or special needs (e.g., elite athletes, pregnant women); or UPF was the outcome not exposure. Studies using non-NOVA food classification systems or non-UPF exposure variables; UPF without definition and cited reference; studies on general food patterns. Conference proceedings, modelling studies, editorials, commentaries, opinions, study protocols, theses, articles where full text was unavailable. Published prior to 2009. Non-English language.

**Table 4:** Diseases and mortality as outcomes.

Study Details			UPF Exposure			Outcomes		Results
Publication Author(s) Year	Study Type (Year) Setting	Population (Number)	Extraction Level	Relative exposure [UPF reference year]	Data Collection Method	Health Outcome	Data Collection Method	Key Findings
**Cancer**								
Queiroz 2018 [81]	Case control study (2015) Brazil	Adult women Mean 53.1 years (*n* = 118)	Individual	UPF ≥ 5 day/week {NOVA.2010] [25]	98-item FFQ, 12-month recall	Breast cancer (BC)	Diagnosed BC	Regular consumption UPF (≥5 day/week) identified as risk factors for BC (OR = 2.35, 95% CI 1.08–5.12).
Fiolet 2018 [84]	Prospective Cohort (2017), 5 years median follow-up France	Adults ≥ 18 years Mean 42.8 (*n* = 104,980)	Individual	UPF % g (quartiles) [NOVA 2018] [30]	3 × 24-h records	Overall, breast, prostate, and colorectal cancer	Self-report or/physician contact	UPF contribution in proportion of grams ranged from 18.7% lowest quartile to 32.3% in highest. A 10% increase in proportion of UPF consumption associated with a significant increase in overall (HR = 1.12; 95% CI 1.06; 1.18; *p* for trend <0.001) and BC risk (HR = 1.11; 95% CI 1.02; 1.22, *P* trend = 0.02). No significant associations were found for prostate and colorectal cancers (*p* = 0.8 and *p* = 0.2, respectively).
**Cardiovascular disease (CVD)**								
Srour 2019 [83]	Prospective cohort (2019), 5.2 years median follow-up France	Adults ≥18 years (*n* = 105,109)	Individual	UPF % grams (quartiles) [NOVA.2018] [30]	3 × 24 h records	Cardiovascular (CVD), coronary heart (CHD), cerebrovascular disease	Medical records, committee of doctors	UPF contribution averaged 17. 4% of total grams. A 10% increase in proportion of UPF consumption was associated with significant higher risk of overall CVD (HR = 1.12; 95% CI 1.05; −1.20, *p* < 0.001); CHD (HR = 1.13; 95% CI 1.10; −1.24, *p* = 0.02); and cerebrovascular disease (HR = 1.11; 95% CI 1.01–1.21; *p* = 0.02).
**Type 2 Diabetes (T2D)**								
Srour 2019 [87]	Prospective cohort (2017), 6.0 years median follow-up France	Adults ≥ 18 Mean 42.7 years (*n* = 104,707)	Individual	UPF % g [NOVA.2018] [30]	3 × 24 h records	Type 2 Diabetes (T2D)	ICD-10 code or T2D medication	Mean UPF contribution was 17.3% by weight, and 29.95% by % E intake. A 10% increase in the proportion of UPF consumption was associated with a significant higher risk of T2D (HR = 1.15; 95% CI 1.06; 1.25; *p* = 0.001).
**Mortality**								
Kim 2019 [94]	Prospective cohort (2011), 19 years median follow-up USA	Adults ≥20 years (*n* = 11,898)	Individual	UPF frequency (times/day) (quartiles) [NOVA.2018] [30]	81-item FFQ, and 24-h recall	All-cause mortality (ACM) CVD mortality	National death index. CVD items 100–169 ICD-10	Participants consumed UPF a mean 4 times/day. Individuals in the highest quartile of frequency of UPF consumption had significantly higher risk of ACM, (HR = 1.31; 95% CI 1.09; 1.58, *p*-trend = 0.001). No significant association was observed with CVD mortality.
Schnabel 2019 [86]	Prospective Cohort (2017), (median follow-up 7.1 years) France	Adults ≥ 45 years (*n* = 44,551)	Individual	UPF % g [NOVA 2018] [30]	3 × 24-h food record	ACM	National death registries. Causes by ICD-10	UPF contributed 14.4% total weight, and 29.9% total E intake. A 10% increase in the proportion of UPF consumption was associated with a significant higher risk of ACM 1.14 (95% CI, 1.04–1.27; *p* = 0.008).
Rico-Campà 2019 [91]	Prospective Cohort (2014), (median follow-up 10.4 years) Spain	Adults 20−91 years (*n* = 19,899)	Individual	UPF servings/day (quartiles) [NOVA.2016] [22]	136-item FFQ	ACM CVD mortality Cancer mortality	Next of kin/Registries	UPF consumption ranged from 1.4 servings a day in lowest quintile to 5.3 servings a day in highest quintile. Individuals in the highest quartile of UPF consumption were at higher risk of ACM (HR = 1.62; 95% CI 1.13; 2.33) than those in the lowest quartile. No significant associations were found for cardiovascular and cancer mortality.
Blanco-Rojo 2019 [92]	Prospective Cohort (2016), (mean follow-up 7.7 years) Spain	Adults Mean 46.9 years (*n* = 11,898)	Individual	UPF % total E intake (quartiles) [NOVA.2018] [30]	880-item FFQ	ACM	National Death Index	UPF contributed 24.4% total E intake. Individuals in the highest quartile of UPF consumption were at higher risk of ACM (HR = 1.46; 95% CI 1.04-2.05; *P* trend = 0.03), than those in the lowest quartile.

Results are presented for adjusted associations for potential confounders and statistically significant associations. NOVA refers to the food classification system [21] or earlier versions, as referenced; UPF: ultra-processed food (includes food and beverages); E:energy in calories or kilojoules; OR: odds ratio; FFQ: food frequency questionnaire; CI: confidence interval; HR: hazards ratio; food = food and beverages; BC = breast cancer; ICD-10:International Classification of Disease; CVDL cardiovascular disease, CHD: coronary heart disease; T2D:Type 2 diabetes; ACM: all-cause mortality.

**Table 5:** Disorders and conditions as outcomes.

Study Details			UPF Exposure			Outcomes		Results
Publication Author(s) Year	Study Type (Year) Setting	Population (Number)	Extraction Level	Relative exposure [UPF Reference Year]	Data Collection Method	Health Outcome (Study Definition)	Data Collection Method	Key Findings
**Gastrointestinal Disease**								
Schnabel 2018 [85]	Cross-sectional (2013) France	Age ≥ 18 years (mean 50.4) (*n* = 33,343)	Individual	UPF % total grams (quartiles) [NOVA.2018] [30]	3 × 24-h records	Functional gastrointestinal disorders (Rome III criteria)	Self-report *	UPF contributed to 16% of total food intake by weight; 33.0% by total E intake. Individuals in the highest quartile of UPF intake had significantly higher risk of IBS (OR = 1.25; 95% CI 1.12; 1.39) and FDy (OR = 1.25; CI 95% 1.05; 1.47) but not FDy alone, compared with those in the lowest quartile.
Vasseur 2020 [88]	Prospective cohort (2016) 2.3 years mean follow-up France	Adults ≥ 18 years (mean 43.3) (*n* = 105,832)	Individual	UPF % total grams (tertiles) [NOVA.2018] [30]	3 × 24 h records	Inflammatory bowel disease	Self-report **	UPF contributed 17% food intake by weight in grams. No significant association was found with UPF consumption and IBD (*p* = 0.03).
**Depression**								
Adjibade 2019 [89]	Prospective Cohort (2012), 5.4 years mean follow-up France	Adults age 18–86 years (n= 26,730)	Individual	UPF % total grams (quartiles) [NOVA.2018] [30]	3 × 24 h records	Depression (CES-D scale)	Self-report *	UPF contributed 5% by weight in grams and 32% E intake. Individuals in the highest quartile of UPF intake had significantly higher risk of developing depressive symptoms (HR = 1.30; 95% CI 1.15–1.47) than those in the lowest quartile. Each 10% increase in UPF consumption was HR of 1.21 (95% CI, 1.15–1.27).
Gomez-Donoso, 2019 [90]	Prospective cohort (2016), 10.3 years median follow-up Spain	Adults (mean 36.7 years) (*n* = 14,907)	Individual	UPF energy adjusted kcal/day (quartiles) [NOVA.2016] [22]	136–item FFQ	Depression	Self-report **	Individuals in highest quartile UPF had significantly higher risk of depression (HR = 1.33; 95% CI 1.07–1.64); *p* trend = 0.004), than individuals in lowest quartile of consumption, after confounder adjustment.
**Frailty**								
Sandoval-Insausti 2019 [93]	Prospective Cohort (2008–2010), 3.5 years median follow-up Spain	Adults ≥ 60 years (*N* = 1822)	Individual	UPF intake % total E (quartiles) [NOVA2018] [30]	Validated interview computerized diet history	Frailty (Fried’s criteria)	Trained personnel	UPF contributed mean of 19.3% total E intake. Individuals in the highest quartile of UPF intake had higher risk of frailty (OR = 3.67; 95% CI 2.00, 6.76) than those in the lowest quartile of intake.
**Asthma (children and adolescents)**								
Melo et al. 2018 [80]	Cross-sectional (2012) Brazil	Grade 9 students (*n* = 109,104)	Individual	UPF score, intake per week (quintiles) [NOVA 2018] [30]	6-UPF sub-categories FFQ	Asthma, wheezing in past 12 months	Self-report *	Individuals in the highest quintile of the UPF intake score had higher odds of having asthma (OR = 1.27; 95% CI 1.15, 1.41) or wheezing (OR = 1.42; 95% CI 1.35 to 1.50), than those in the lowest quintile.
Azeredo 2020 [82]	Prospective Cohort (2004–2010) Brazil	Children mean age 6.8 years baseline; 11.0 years at follow-up (*n* = 2190)	Individual	UPF % total E intake (quintiles) [NOVA.2018] [30]	55 (age 6) and 88 items (age 11) FFQ.	Wheezing, whistling or asthma in past 12 months	Self-report *	UPF contribution to total E intake was 42.3% at 6 years, and 33.7% at 11 years. Consumption of UPF at age 6 was not significantly associated with wheeze, asthma or severe asthma at age 11.

Results are presented for adjusted associations for potential confounders and statistically significant associations. NOVA refers to the food classification system [21] or earlier versions, as referenced; food means foods and beverages; * self-report from questionnaire on condition, medical history, symptoms, medication use, and/or diagnosis by medical practitioner; ** questionnaire plus validation on sample laboratory test or interview; UPF: ultra-process food (includes food and beverages); E: energy in calories or kilojoules; OR: odds ratio; CI: confidence interval; IBS: irritable bowel syndrome; FDy: functional dyspepsia; IBD: inflammatory bowel disease; CES-D scale: Centre for Epidemiologic Studies Depression Scale; HR: hazards ratio; FFQ: food frequency questionnaire; HR: hazards ratio.

## Supplementary Materials

The following are available online at https://www.mdpi.com/2072-6643/12/7/1955/s1, Table S1: NOVA food groups definitions according to the extent and purpose of food processing, with examples; Table S2: NOVA ultra-processed food sub-groups reported in references in studies. Table S3: Quality assurance process.

## Abbreviations

BMI	Body Mass Index
CRP	C-reactive protein
CVD	Cardiovascular disease
DALY	Disability life adjusted years
FFQ	Food frequency questionnaire
GBD	Global burden of disease
HDL	High-density lipoprotein cholesterol
IBS	Irritable bowel syndrome
LDL	Low density lipoprotein cholesterol
NCD	Non-communicable disease
MPF	Unprocessed and minimally processed foods
NHANES	National Health and Nutrition Examination Survey
PCI	Processed culinary ingredients
PF	Processed foods
SSB	Sugar-sweetened beverages
UPF	Ultra-processed foods
WHO	World Health Organization
