# Supplementary material for: Ultra-Processed Foods and Health Outcomes: A Narrative Review
Source: Nutrients. 2020 Jun 30;12(7):1955. doi: 10.3390/nu12071955 (PMC7399967; doi:10.3390/nu12071955)
Supplement: Supplementary file 1 [file nutrients-12-01955-s001.pdf]

Ultra-processed foods and health outcomes: a narrative review

Leonie Elizabeth, Priscila Machado, Marit Zinöcker, Phillip Baker, Mark Lawrence

SUPPLEMENTARY MATERIALS:

Supplementary Table S1: NOVA Food Groups definitions according to the extent and purpose of food processing, with examples

Supplementary Table S2: NOVA ultra-processed food sub-groups reported in references in studies

Supplementary Table S3: Quality assurance process

Supplementary Table S1

NOVA food groups: definitions according to the extent and purpose of food processing, with examples*

NOVA group	Definition	Examples
**1) Unprocessed or minimally processed foods**	Unprocessed: edible parts of plants (fruits, seeds, leaves, stems, roots, tubers) or of animals (muscle, offals, eggs, milk), and also fungi, algae and water, after separation from nature.<br><br>Minimally processed: unprocessed foods altered by industrial processes such as removal of inedible or unwanted parts, drying, crushing, grinding, fractioning, roasting, boiling, pasteurisation, refrigeration, freezing, placing in containers, vacuum packaging, non-alcoholic fermentation, and other methods that do not add salt, sugar, oils or fats or other food substances to the original food. The main aim of these processes is to extend the life of unprocessed foods, enabling their storage for longer use, and, often, to make their preparation easier or more diverse. Infrequently, minimally processed foods contain additives that prolong product duration, protect original properties or prevent proliferation of microorganisms.	Fresh, squeezed, chilled, frozen, or dried fruits and leafy and root vegetables; grains such as brown, parboiled or white rice, corn cob or kernel, wheat berry or grain; legumes such as beans, lentils, and chickpeas; starchy roots and tubers such as potatoes, sweet potatoes and cassava; fungi such as fresh or dried mushrooms; meat, poultry, fish and seafood, whole or in the form of steaks, fillets and other cuts, fresh or chilled or frozen; eggs; fresh or pasteurized milk; fresh or pasteurised fruit or vegetable juices (with no added sugar, sweeteners or flavours); grits, flakes or flour made from corn, wheat, oats, or cassava; tree and ground nuts and other oily seeds (with no added salt or sugar); herbs and spices used in culinary preparations, such as thyme, oregano, mint, pepper, cloves and cinnamon, whole or powdered, fresh or dried; fresh or pasteurized plain yoghurt; tea, coffee, and drinking water. Also includes foods made up from two or more items in this group, such as dried mixed fruits, granola made from cereals, nuts and dried fruits with no added sugar, honey or oil; pasta, couscous and polenta made with flours, flakes or grits and water; and foods with vitamins and minerals added generally to replace nutrients lost during processing, such as wheat or corn flour fortified with iron and folic acid.
**2) Processed culinary ingredients**	Substances obtained directly from group 1 foods or from nature by industrial processes such as pressing, centrifuging, refining, extracting or mining. Their use is in the preparation, seasoning and cooking of group 1 foods. These products may contain additives that prolong product duration, protect original properties or prevent proliferation of microorganisms.	Vegetable oils crushed from seeds, nuts or fruits (notably olives); butter and lard obtained from milk and pork; sugar and molasses obtained from cane or beet; honey extracted from combs and syrup from maple trees; starches extracted from corn and other plants, and salt mined or from seawater, vegetable oils with added anti-oxidants, and table salt with added drying agents. Includes products consisting of two group 2 items, such as salted butter, and group 2 items with added vitamins or minerals, such as iodised salt.
**3) Processed foods**	Products made by adding salt, oil, sugar or other group 2 ingredients to group 1 foods, using preservation methods such as canning and bottling, and, in the case of breads and cheeses, using non-alcoholic fermentation. Processes and ingredients here aim to increase the durability of group 1 foods and make them more enjoyable by modifying or enhancing their sensory qualities. These products may contain additives that prolong product duration, protect original properties or prevent proliferation of microorganisms.	Canned or bottled vegetables and legumes in brine; salted or sugared nuts and seeds; salted, dried, cured, or smoked meats and fish; canned fish (with or without added preservatives); fruits in syrup (with or without added anti-oxidants); freshly made unpackaged breads and cheeses.

**4) Ultra-processed foods**	Formulations of ingredients, mostly of exclusive industrial use, that result from a series of industrial processes (hence 'ultra-processed'), many requiring sophisticated equipment and technology. Processes enabling the manufacture of ultra-processed foods include the fractioning of whole foods into substances, chemical modifications of these substances, assembly of unmodified and modified food substances using industrial techniques such as extrusion, moulding and pre-frying, frequent application of additives whose function is to make the final product palatable or hyper-palatable ('cosmetic additives'), and sophisticated packaging, usually with synthetic materials. Ingredients often include sugar, oils and fats, and salt, generally in combination; substances that are sources of energy and nutrients but of no or rare culinary use such as high fructose corn syrup, hydrogenated or interesterified oils, and protein isolates; cosmetic additives such as flavours, flavour enhancers, colours, emulsifiers, sweeteners, thickeners, and anti-foaming, bulking, carbonating, foaming, gelling, and glazing agents; and additives that prolong product duration, protect original properties or prevent proliferation of microorganisms. Processes and ingredients used to manufacture ultra-processed foods are designed to create highly profitable products (low cost ingredients, long shelf-life, emphatic branding), convenient (ready-to-consume) hyper-palatable snacked products liable to displace all other NOVA food groups, notably group 1 foods.	Carbonated soft drinks; sweet or savoury packaged snacks; chocolate, candies (confectionery); ice-cream; mass-produced packaged breads and buns; margarines and other spreads; cookies (biscuits), pastries, cakes, and cake mixes; breakfast 'cereals', 'cereal' and 'energy' bars; 'energy' drinks; milk drinks, 'fruit' yoghurts and 'fruit' drinks; 'cocoa' drinks; 'instant' sauces; infant formulas, follow-on milks, other baby products; 'health' and 'slimming' products such as meal replacement shakes and powders. Many ready to heat products including pre-prepared pies and pasta and pizza dishes; poultry and fish 'nuggets' and 'sticks', sausages, burgers, hot dogs, and other reconstituted meat products, and powdered and packaged 'instant' soups, noodles and desserts.

* Alcoholic drinks are not immediately classifiable by NOVA. By analogy with the nature of processed and ultra-processed foods, they may be counted in group 3 if they are produced by fermentation of group 1 foods, such as beer, cider, and wine, and in group 4 if they are produced by fermentation of group 1 foods and distillation of the resulting alcohol, such as whisky, gin, rum, and vodka. Another option, depending on why NOVA is being used, is to treat alcoholic drinks separately.

Reproduced with permission.

Reference: [1] Monteiro, CA; Cannon, G; levy, RB; Moubarac, J-C; Louzada, MLC; Rauber, F; Khandpur, N; Cediel, G; Neri, D; Matrinez-Steele, E; Baraldi, LG; Jaime, PC. *Ultra-processed foods: What they are and how to identify them*. *Public Health Nutrition*. **2019.** 22 (5) 936-941. Supplemental Table S 1.

Supplementary Table S2: Ultra-processed food sub-groups reported in references in studies

	Year of NOVA reference									
**UPF sub-categories included NOVA-2019**	**NOVA-2019**	**NOVA-2009**	**NOVA-2010**	**NOVA-2012**	**NOVA-2014**	**DGB-2014**	**NOVA-2016a**	**NOVA-2016b**	**NOVA-2017**	**NOVA-2018**
(Reference)	[1]	[2]	[3]	[4]	[5]	[6]	[7]	[8]	[9]	[10]
Fish Nuggets, chicken nuggets	●	●	●	●	●	●	●	●	●	●
Hot Dogs	●	●	●	●	●	●	●		●	●
Burgers	●	●	●	●	●	●	●	●	●	●
Fish sticks / sticks (seafood imitation)	●		●	●		●	●	●	●	●
Sausages (luncheon meats, beef jerky)	●	●	●			●	●		●	●
Reconstituted meat (extruded meat, meat remnants)	●	●		●		●	●	●	●	●
Mass produced bread and buns *	●			●	●	●	●	●	●	●
Breakfast cereals (morning cereals)(+/- 'added sugar)	●	●	●	●	●	●	●	●	●	●
Cakes (base of flour), (cake mixes, crepes)	●		●	●	●	●	●	●	●	●
Cookies (biscuits); savoury biscuits	●	●	●	●	●	●	●	●	●	●
Cereal bars (energy bars, granola bars, protein bars)	●	●	●	●	●	●	●		●	●
Pastry, pastries	●		●	●	●		●	●	●	●
Desserts / frozen desserts (puddings, dry mix desserts)	●			●	●		●	●	●	●
Frozen / pre-prepared pasta dishes	●		●	●	●	●	●		●	●
Frozen / pre-prepared pizza dishes (pies)(bakery products)	●		●	●	●	●	●	●	●	●
Canned/dehydrated/powder soups, stews, instant noodle	●		●	●	●	●	●	●	●	●
Confectionary (candy, sweets, pastilles)	●	●	●	●	●	●	●		●	●
Chocolate	●	●	●	●	●		●		●	●
(Packaged) Sweet snack foods	●	●	●	●	●	●	●	●	●	●
Ice-cream (and ice pops)	●	●	●	●	●	●	●	●	●	●
Sweetened / frozen / fruit yoghurt / flavoured yoghurt	●				●	●	●	●	●	●
Savoury snack foods (fatty or salty)(+/- popcorn)	●	●	●	●	●	●	●	●	●	●
Chips (crisps)	●	●	●	●	●			●		
Sugared fruir and fruit nectar drinks (juice powders)	●		●	●	●	●	●	●	●	●
Cocoa drinks (chocolate drinks)	●				●		●		●	●
Carbonated sugared drinks, other soft drinks, soda	●	●	●	●	●	●		●	●	●
Energy drinks (sports drinks not always mentioned)	●			●	●	●	●	●	●	●
Sugared milk drinks (dairy drinks)(milk shakes, malted milk)	●		●	●	●	●	●	●	●	●
Sauces, mayonnaise (includes instant sauces dressings)	●		●	●	●		●	●	●	●
Margarines **	●			●	●		●	●	●	●
Other Spreads (dips)	●						●	●	●	●
Infant formulas	●		●	●	●		●	●	●	●
Follow-on milks	●		●	●	●		●		●	●
Baby food/products (including rusks)	●		●	●	●		●	●	●	●
Health products	●				●		●		●	●
Slimming products	●				●		●		●	●
Meal replacements; fortified meal and dish substitutes	●				●		●		●	●
pre-frying	●									

Supplementary Table S2 (continued)

	NOVA References										
**UPF sub-categories not included NOVA-2019**	**NOVA-2019**	**NOVA-2009**	**NOVA-2010**	**NOVA-2012**		**NOVA-2014**	**DGB-2014**	**NOVA-2016a**	**NOVA-2016b**	**NOVA-2017**	**NOVA-2018**
Sub-groups intermittently included as UPF	(1)	(2)	(3)	(4)		(5)	(6)	(7)	(8)	(9)	(10)
Pre-prepared meat, poultry, fish, vegetable dishes			●	●		●	●		●		
Pre-prepared cheese dishes, (cheese products)				●		●					
Chips (French fries)			●	●		●			●		
Condensed milk						●					
Ready-to-drink tea and coffee (sweetened)									●		
Meat, yeast extracts (chicken extracts)				●		●		●	●	●	●
Processed cheese											●
Jam / preserves / fruit sauces			●	●		●					
Previously classed as UPF, now classed MPF, PCI or PF											
Instant coffee #						●					
Vegetables canned or bottled in brine ξ			●								
Salted, smoked, pickled, cured meat or fish (ham, bacon) ξ		●	●								
Fish canned in oil ξ			●								
Cheeses ξ			●								
Artisanal bread (UPF includes all bread in early studies) ξ		●	●	●		●			●		
Fruit canned in syrup ξ			●								

Key:
Grey shaded areas are NOVA-2019 Group 4 (UPF) sub-groups (Reference for this review)
UPF: ultra-processed foods; MPF: minimally processed foods; PF: processed foods; PCI: processed culinary ingredients

Dots are sub-categories included in references listed by authors

*Bread that includes hydrogenated vegetable fat, whey, emulsifiers, other additives (mass produced)(buns).

** Margarine previously PCI, now UPF

Previously UPF
ξ Previously UPF, now PF

UPF: ultra-processes food, MPF: unprocessed or minimally processed foods, PCI: processed culinary ingredients, PF: processed foods

Supplementary Table S 3: Quality Assurance Process:

A quality assurance process was undertaken to build confidence in findings and conclusions.

**Step**	**Quality Assurance Detail**
1. Methods	Methods were developed by all authors with decisions reached by consensus.
2. Search	Systemised search was conducted by LE after discussions with ML and expert librarian.
3. Screening	Initial screening of titles and abstracts by LE of our inclusion/ exclusion criteria (as listed in Table 1) ensured papers met minimum criteria for a research question, publication and study details, target group, exposure and outcome. Queries on inclusions were discussed with ML. Any paper still in doubt was taken to all authors for discussion until consensus reached.
4.Data extraction and tabulation	Relevant data was extracted to Tables by LE. PM reviewed table and extracted data on key findings. All authors reviewed final tables for data extracted, statistics, and key findings.
5. Quality Assessment	A Quality assessment was conducted by LE using tools appropriate for each type of study: NIH [11] for cross-sectional and prospective cohort studies; SIGN for case-control study [12]; and CASP for random-controlled trial [13]; with amendments of questions, where applicable to NOVA. A selection of studies were independently reviewed by a second author (ML, MKZ or PM). For discrepancies between the two authors, it was taken to a third reviewer, or discussed by all authors until consensus was reached. The purpose of the quality assessment process was to identify limitations within studies. It was not to assign an overall quality-rating for any particular study. Limitations were noted for studies that did not have:<br>• clearly stated aim(s) and objectives, well defined and appropriate method, a clear statement of results, and conclusions consistent with the study findings;<br>• population clearly specified, sample representative of population, sample size justification given, and (for cohort studies) loss to follow-up 20% or less;<br>• UPF exposure measurement before outcome measurement; with outcome assessor blinded to exposure status; and (for prospective cohort studies) exposure measured more than once over time;<br>• exposure level comparison;<br>• NOVA application detailed;<br>• food data extraction by appropriate reliable validated measure;<br>• outcome measures by validated reliable method; trained personnel; medical records; or (for population studies) national registries or statistical records;<br>• key confounders considered and appropriate statistics applied;<br>• (for case-control study) cases and controls comparable populations; clearly differentiated; same exclusions applied; similarities and differences stated;<br>• (for random-controlled trial) patients randomised; health workers, study participants, study personnel “blinded” to treatment; groups treated equally except for intervention; precise estimate of treatment effect;

References:

1. Monteiro, C.A.; Cannon, G.; Levy, R.B.; Moubarac, J.C.; Louzada, M.L.; Rauber, F.; Khandpur, N.; Cediel, G.; Neri, D.; Martinez-Steele, E., et al. Ultra-processed foods: What they are and how to identify them. *Public Health Nutr* **2019**, *22*, 936–941, doi:10.1017/S1368980018003762.
2. Monteiro, C.A. Nutrition and health. The issue is not food, nor nutrients, so much as processing. *Public Health Nutr* **2009**, *12*, 729–731, doi:10.1017/S1368980009005291.
3. Monteiro, C.A.; Levy, R.B.; Claro, R.M.; de Castro, I.R.R.; Cannon, G. A new classification of foods based on the extent and purpose of their processing. *Cad Saude Publica* **2010**, *26*, 2039–2049, doi:10.1590/S0102-311X2010001100005.
4. Monteiro, C.A.; Cannon, G.; Levy, R.; Claro, R.; Moubarac, J.-C. The food system. Ultra-processing: The big issue for nutrition, disease, health, well-being. [Commentary]. *World Nutr* **2012**, *3*, 527–569.
5. Moubarac, J.C.; Parra, D.C.; Cannon, G.; Monteiro, C.A. Food Classification Systems Based on Food Processing: Significance and Implications for Policies and Actions: A Systematic Literature Review and Assessment. *Curr Obes Rep* **2014**, *3*, 256-272, doi:10.1007/s13679-014-0092-0.
6. Ministry of Health of Brazil, Secretariat of Health Care, Primary Health Care Department. *Dietary Guidelines for the Brazilian Population, 2nd ed.*; Ministry of Health Brazil: Brasilia, 2014.
7. Monteiro, C.; Cannon, G.; Levy, R.; Moubarac, J.C.; Jaime, P.; Martins, A.P.; Canella, D.; Louzada, M.L.; Parra, D. NOVA. The star shines bright. Food classification. Public Health. *World Nutr J* **2016**, *7*, 28–38.
8. Martínez Steele, E.; Baraldi, L.G.; da Costa Louzada, M.L.; Moubarac, J.C.; Mozaffarian, D.; Monteiro, C.A. Ultra-processed foods and added sugars in the US diet: Evidence from a nationally representative cross-sectional study. *BMJ Open* **2016**, *6*, e009892–e009892, doi:10.1136/bmjopen-2015-009892.
9. Moubarac, J.C.; Batal, M.; da Costa Louzada, M.L.; Martinez Steele, E.; Monteiro, C.A. Consumption of ultra-processed foods predicts diet quality in Canada. *Appetite* **2017**, *108*, 512–520, doi:10.1016/j.appet.2016.11.006.
10. Monteiro, C.A.; Cannon, G.; Moubarac, J.C.; Levy, R.B.; Louzada, M.L.C.; Jaime, P.C. The UN Decade of Nutrition, the NOVA food classification and the trouble with ultra-processing. *Public Health Nutr* **2018**, *21*, 5–17, doi:10.1017/S1368980017000234.
11. National Heart Lung and Blood Institute. Quality assessment tool for observational cohort and cross-sectional studies. [online]. **2020**, Available online: https://www.nhlbi.nih.gov/health-topics/study-quality-assessment-tools, (accessed 28 January, 2020).
12. Scottish Intercolleiate Guidelines Network. SIGN methodology checklist: Case-control studies. [online]. **2012**, Available online: https://www.sign.ac.uk/checklists-and-notes (accessed 28 January, 2020).
13. Critical Appraisal Skills Programme. CASP Randomised Controlled Trial checklist. [online]. **2018**, Available online: https://casp-uk.net/wp-content/uploads/2018/2001/CASP-Qualitative-Checklist-2018.pdf, (accessed January, 2020).
